# Haemagglutinin substitutions N125D, D127E, D222G and R223Q improve replicative fitness and vaccine effectiveness of an A/H1N1pdm09 live attenuated influenza vaccine virus by enhancing α-2,6 receptor binding

**DOI:** 10.1371/journal.ppat.1010585

**Published:** 2022-05-27

**Authors:** Rachael Dempsey, Giulia Tamburrino, Katarzyna E. Schewe, Jonathan Crowe, Annalisa Nuccitelli, Oliver Dibben

**Affiliations:** 1 Flu-BPD, Biopharmaceuticals R&D, AstraZeneca, Liverpool, United Kingdom; 2 *In vivo* expressed biologics, Discovery Sciences, R&D, AstraZeneca, Cambridge, United Kingdom; Chang Gung University, TAIWAN

## Abstract

During 2013–14 and 2015–16, A/H1N1pdm09 live attenuated influenza vaccine (LAIV) viruses replicated inefficiently in primary human nasal epithelial cells (hNEC). This led to reduced vaccine effectiveness (VE) in quadrivalent formulations, mediated by inter-strain competition. By mutating the haemagglutinin (HA) protein, we aimed to enhance hNEC replication of a novel A/H1N1pdm09 vaccine strain to overcome competition and improve VE. Combinations of N125D, D127E, D222G and R223Q substitutions were introduced to the HA protein of A/Slovenia/2903/2015 (A/SLOV15). A/SLOV15 S13, containing all four HA substitutions, produced approximately 1000-fold more virus than parental V1 during hNEC infection. Immunogenicity in ferrets was increased by approximately 10-fold, without compromising yield in eggs or antigenic match to wild-type (*wt*) reference strains. Despite S13 and V1 being antigenically similar, only S13 protected ferrets from *wt* virus shedding and fever post-challenge. Crucially, these data suggested that enhanced fitness allowed S13 to overcome inter-strain competition in quadrivalent LAIV (QLAIV). This improved efficacy was later validated by real-world VE data. S13 displayed increased binding avidity to a mammalian-like α-2,6 receptor analogue (6-SLN), relative to V1, while maintaining avian-like 3-SLN avidity. *In silico* modelling of the HA receptor binding site revealed additional interactions in the S13:6-SLN binding network and a mild increase in 6-SLN binding energy, indicating a possible mechanism for increased α-2,6 receptor-binding avidity. These data confirm that rational HA mutagenesis can be used to optimise hNEC replication and VE for A/H1N1pdm09 LAIV viruses.

## Author summary

Vaccine effectiveness for the pandemic H1N1 component of the FluMist/Fluenz live attenuated influenza vaccine was lower than expected during the 2013–14 and 2015–16 influenza seasons in the USA. Previous studies highlighted the importance of vaccine viruses replicating sufficiently well in human cells that they could compete in quadrivalent formulations and protect ferrets against influenza-like illness.

Here, it was demonstrated that mutagenesis of the haemagglutinin surface protein can be used to improve vaccine effectiveness of a pandemic H1N1 vaccine strain. This was achieved by optimising replication in human cells while, crucially, leaving other key characteristics required for vaccine production unaffected. The mutated vaccine virus successfully overcame inter-strain competition, providing protection from influenza-like illness in ferrets, while its parent did not. Furthermore, it was demonstrated that enhanced binding to human-like receptors was the mechanism by which replicative fitness was being improved.

These data suggested that improving replicative fitness, allowing the vaccine virus to drive a protective immune response in quadrivalent formulations, would result in increased effectiveness in patients. Subsequently, high vaccine effectiveness was demonstrated during the 2017–18 northern hemisphere influenza season, validating this approach.

## Introduction

Influenza is a significant cause of morbidity and mortality worldwide, with seasonal epidemics resulting in ~3–5 million severe infections and ~290,000–650,000 deaths annually [1]. Consequently, controlling seasonal and pandemic influenza outbreaks is a major global public health concern, with annual vaccination being the most effective way to reduce the spread of infection [1–3]. A quadrivalent live attenuated influenza vaccine (QLAIV), FluMist/Fluenz, was licensed for use in the United States of America (USA) in 2012 and the European Union (EU) in 2013 [4,5]. It was originally licensed in a trivalent formulation in the USA in 2003. QLAIV contains representative pandemic 2009 H1N1 (A/H1N1pdm09), A/H3N2, B-Victoria and B-Yamagata LAIV strains that are generated by reverse genetics [6]. The surface glycoprotein genes, haemagglutinin (HA) and neuraminidase (NA), from currently circulating wild-type (*wt*) influenza strains, are reassorted with genes derived from the cold adapted (*ca*), temperature sensitive (*ts*) and attenuated (*att*) master donor virus, A/Ann Arbor/6/1960 [7–9] for A-strains or B/Ann Arbor/1/1966 for B-strains [10–12], to produce candidate vaccine viruses (CVVs). LAIV is administered intranasally and relies on the replication of vaccine viruses in the upper respiratory tract, resulting in both humoral and cell-mediated immune responses via serum antibodies, mucosal antibodies and influenza-specific T-cells [13,14].

During the 2013–14 and 2015–16 influenza seasons, the A/H1N1pdm09 components of QLAIV, A/California/07/2009 (A/CA09) and A/Bolivia/559/2013 (A/BOL13), respectively, were found to have reduced vaccine effectiveness (VE) in the USA [15–17]. A/H1N1pdm09 LAIV strains were found to have reduced replicative fitness in fully differentiated primary human nasal epithelial cells (hNEC), relative to pre-pandemic H1N1 viruses, identifying a potential root cause for reduced A/H1N1pdm09 VE [18]. The critical contribution of A/H1N1pdm09 fitness to VE was then confirmed *in vivo* using an optimised ferret challenge model, which demonstrated that the reduced fitness of A/BOL13 led to inter-strain competition and loss of efficacy in multivalent vaccine formulations [19].

To improve on the reduced VE of A/BOL13, the A/H1N1pdm09 component of FluMist/Fluenz required updating to a strain with enhanced replicative fitness in hNEC for the 2017–18 season. To achieve this, the use of rational mutagenesis of the HA protein was explored.

While HA protein mutagenesis had been used successfully to enhance egg yield in the past [20,21], optimisation of HA sequences to enhance LAIV replication in human cells and, consequently VE, had never been studied in development of a LAIV CVV. FluMist/Fluenz is manufactured in embryonated hens eggs, thus hNEC replication would require optimisation without compromising egg yield. In addition, the potential impact of this approach on factors such as LAIV antigenicity or HA thermostability, which has previously been implicated in A/H1N1pdm09 VE [22–24], needed to be studied as well.

A representative of the newly emerged 6B.1 clade, A/Slovenia/2903/2015 (A/SLOV15), was chosen to undergo HA mutagenesis to optimise replicative fitness in hNEC. Four amino acid substitutions were selected for introduction into the HA protein of A/SLOV15: N125D, D127E, D222G and R223Q. These substitutions were initially identified in LAIV as minor quasispecies during vaccine development of egg-grown A/H1N1pdm09 strains [20,21,25]. The 125 residue sits within the Sa antigenic site [26], and both the 125 and 127 residues are positioned on the surface of the HA globular head in close proximity to the receptor binding site (RBS) [21]. N125D and D127E residues were shown to enhance LAIV yield in eggs and MDCK cells [21]. However, due to the limited ability of MDCK cells to predict hNEC replication [18], their role in human cell fitness was unknown. The 222 and 223 residues, located within the RBS, are known to play a role in HA receptor binding specificity [20,27–29]. D222G has been shown to confer dual α-2,3 and α-2,6 sialic acid specificity [27,29], as well as increased replication in human bronchial epithelial cells and the upper respiratory tracts of mice and ferrets [28]. A/H1N1pdm09 circulating *wt* strains carrying the D222G substitution were also associated with increased disease severity during the 2009 influenza pandemic [29–31]. Restoration of α-2,6 binding capability, as well as a modest reduction in α-2,3 binding specificity has been observed for viruses containing the R223Q substitution [27]. We hypothesised that incorporation of combinations of these four residues into A/SLOV15 could enhance replication in hNEC, leading to improved VE relative to A/BOL13, without significantly limiting egg yield.

Here we used HA mutagenesis to optimise the replication of A/SLOV15 in hNEC, while maintaining sufficient egg yield for LAIV manufacture and without compromising virus immunogenicity or antigenicity. We aimed to demonstrate that by optimising replicative fitness in human cells, A/SLOV15 would overcome the inter-strain interference observed for A/BOL13 [19] and prove efficacious as a component in QLAIV in ferrets, leading to increased VE in the clinic. In addition, we set out to show that binding to α-2,6 linked sialic acid receptors had been preferentially enhanced, providing a clear mechanism for the improvement of replicative fitness of A/SLOV15 in human cells. This could then be applied to development of high VE A/H1N1pdm09 CVVs in future seasons.

## Results

### A/SLOV15 mutants demonstrated enhanced replicative fitness in hNEC relative to A/SLOV15 V1

Site directed mutagenesis was performed on the egg-derived *wt* A/SLOV15 HA protein to generate a panel of A/SLOV15 LAIV mutants carrying between 1–5 amino acid changes relative to *wt* (Table 1). N125D, D127E, D222G and R223Q were engineered into the A/SLOV15 HA protein as we hypothesised that these substitutions would improve replicative fitness, while the N380D substitution in mutant S13 was identified in a naturally occurring A/SLOV15 variant. Replicative fitness of all variants in human cells was assessed by infecting hNEC at a multiplicity of infection (MOI) of 0.01 50% Tissue Culture Infectious Dose (TCID_50_)/cell and collecting apical washes daily during a five day time-course. Samples were taken from nine transwells per virus, across 3 independent experiments and titrated by TCID_50_. Replication of A/SLOV15 LAIV viruses in hNEC was expressed as the mean daily virus production from each hNEC transwell over five days of infection, as a measure of each mutant’s ability to establish sustained virus replication. This generated a single data point per transwell for statistical analysis, while still utilising all time-course data as described previously [19,32] (shown in full in S1 Fig).

**Table 1:** Summary of A/SLOV15 LAIV mutant HA sequences.

	HA sequence					Identifier
	125	127	222	223	380	
Egg-derived *wt* A/SLOV15 reference	N	D	D	R	N	
A/SLOV15 V1						V1
A/SLOV15 N125D	D					S2
A/SLOV15 D127E		E				S3
A/SLOV15 D222G			G			S4
A/SLOV15 R223Q				Q		S5
A/SLOV15 N125D, D127E	D	E				S6
A/SLOV15 D127E, D222G		E	G			S7
A/SLOV15 D127E, R223Q		E		Q		S8
A/SLOV15 D222G, R223Q			G	Q		S9
A/SLOV15 N125D, D127E, R223Q	D	E		Q		S10
A/SLOV15 D127E, D222G, R223Q		E	G	Q		S11
A/SLOV15 N125D, D127E, D222G, R223Q	D	E	G	Q		S12
A/SLOV15 N125D, D127E, D222G, R223Q, N380D	D	E	G	Q	D	S13

Summary table outlining the HA sequences produced by site-directed mutagenesis of the egg-derived *wt* A/SLOV15 HA. A/SLOV15 V1 has the *wt* HA reference sequence and mutations were introduced stepwise into V1 to optimise hNEC replication. Mutations at residues 125, 127, 222 and 223 were engineered into the HA protein by site-directed mutagenesis, while the N380D substitution was included in S13 as an additional change that was found in a naturally occurring A/SLOV15 variant. All LAIV variants contained the *wt* A/SLOV15 NA gene. A/SLOV15 V1 and the panel of mutants were given identifiers (righthand column) for simplicity.

**Fig 1:**
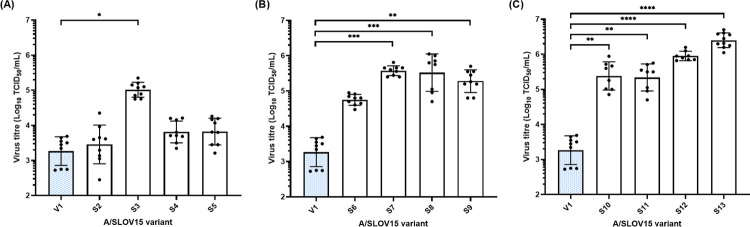
HA mutations provide a cumulative increase in A/SLOV15 replication in primary human nasal epithelial cells (hNEC). Five day time-course infections in hNEC were conducted at an MOI of 0.01. Apical wash samples were collected every 24 hours and virus titre measured by TCID_50_ assay. Mean virus titre per day was calculated from three independent experiments of three transwells each (total nine transwells per virus). Mean daily virus titre of A/SLOV15 V1 (blue) and A/SLOV15 variants carrying: (A) a single HA mutation relative to the *wt* HA sequence; (B), two HA mutations relative to *wt;* and (C) 3–5 mutations relative to *wt* are illustrated by the columns. Symbols depict mean daily virus titre for individual hNEC transwells while columns show group means and error bars indicate the standard deviation. A Kruskal-Wallis test with a post-hoc Dunn’s multi-comparison test was conducted to compare A/SLOV15 V1 to all other A/SLOV15 variants. P values indicating significant difference in hNEC replication relative to A/SLOV15 V1 are depicted in panels A-C as follows: **** P<0.0001, *** P<0.001, ** P<0.01 and * P<0.05. A/SLOV15 mutants S2-S13 were generated by introducing the following substitutions to *wt* A/SLOV15 HA (V1) sequence: S2: N125D; S3: D127E; S4: D222G; S5: R223Q; S6: N125D, D127E; S7: D127E, D222G; S8: D127E, R223Q; S9: D222G, R223Q; S10: N125D, D127E, R223Q; S11: D127E, D222G, R223Q; S12: N125D, D127E, D222G, R223Q; S13: N125D, D127E, D222G, R223Q, N380D. All variants contained *wt* A/SLOV15 NA sequence.

TCID_50_ titres obtained for A/SLOV15 mutants S2-S13 were compared to the parental V1, which carried the *wt* HA sequence (Fig 1). Minimal improvement was observed in hNEC replication for A/SLOV15 S2, S4 and S5, which carried the single N125D, D222G or R223Q mutations, relative to A/SLOV15 V1 and these differences were not found to be statistically significant (Fig 1A). However, there was a statistically significant increase in hNEC replication for S3, which carried the D127E single mutation (P < 0.05), replicating to a mean titre of 5.01 Log_10_TCID_50_/mL per day during the five day time-course, relative to 3.27 Log_10_TCID_50_/mL per day for V1. The N125D, D127E double mutant (mutant S6) also replicated to higher titres in hNEC than V1, with a mean virus titre of 4.75 Log_10_TCID_50_/mL per day over five days, but this was not statistically significant (Fig 1B). All remaining A/SLOV15 variants, S7-13, which carried two or more mutations, replicated to significantly higher titres in hNEC relative to A/SLOV15 V1 (Fig 1B and 1C). However, the most notable improvement in hNEC replication was observed for A/SLOV15 S13, with virus production being 1000-fold higher than A/SLOV15 V1 over the five day time-course (Fig 1C, P < 0.0001). In summary, these data demonstrated that HA mutagenesis can be used to improve hNEC replication of A/H1N1pdm09 LAIV viruses.

### Virus yield in embryonated hen’s eggs was not compromised by improved replication in hNEC

Virus yield in embryonated hen’s eggs was assessed by inoculating A/SLOV15 LAIV variants at 125 TCID_50_/egg. Nine eggs were inoculated per timepoint across three independent experiments. Eggs were harvested at 24h, 48h, 72h and 90h post-infection and virus present in the allantoic fluid titrated by TCID_50_ assay. Yield in eggs was then expressed as geometric mean of peak TCID_50_ titre obtained for each virus during the three independent experiments (shown in full in S2 Fig). This indicated whether mutants reached suitable peak egg titres for the LAIV manufacturing process.

**Fig 2:**
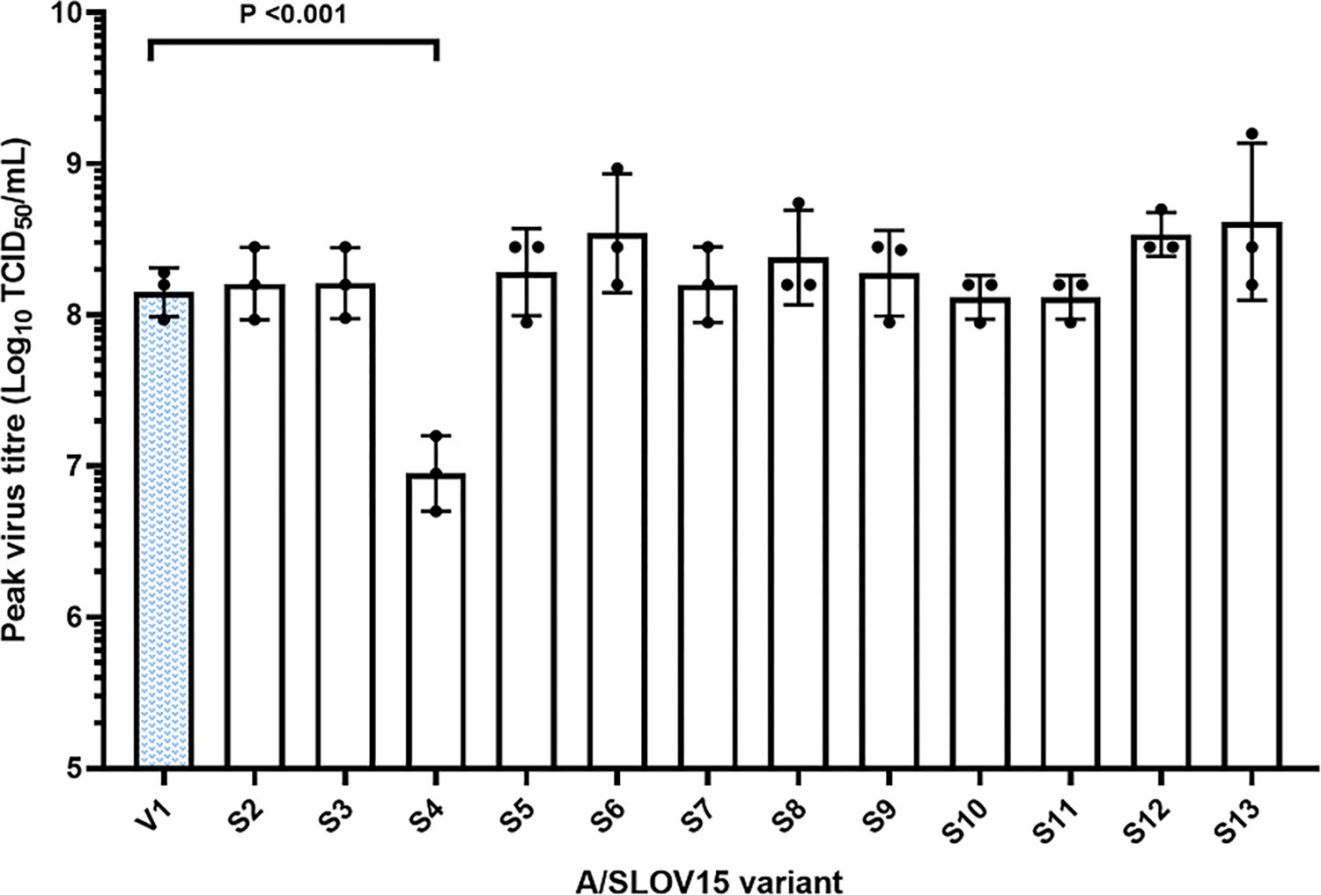
Replication in embryonated hen’s eggs is comparable for A/SLOV15 variants. Embryonated hens eggs were inoculated with 125 TCID_50_/egg of each A/SLOV15 LAIV, and virus yield was measured by TCID_50_ assay of allantoic fluid collected every 24-hours post-infection. Mean peak titre achieved by each A/SLOV15 variant across three independent experiments is expressed by the columns. Symbols depict the individual peak titres from each experiment and error bars indicate standard deviation. A/SLOV15 V1 is shown in blue. One-way ANOVA with a Dunnett’s multi-comparison test was conducted to compare A/SLOV15 V1 to all other A/SLOV15 variants. Significant differences are indicated by P values. A/SLOV15 mutants S2-S13 were generated by introducing the following substitutions to *wt* A/SLOV15 HA (V1) sequence: S2: N125D; S3: D127E; S4: D222G; S5: R223Q; S6: N125D, D127E; S7: D127E, D222G; S8: D127E, R223Q; S9: D222G, R223Q; S10: N125D, D127E, R223Q; S11: D127E, D222G, R223Q; S12: N125D, D127E, D222G, R223Q; S13: N125D, D127E, D222G, R223Q, N380D. All variants contained *wt* A/SLOV15 NA sequence.

To evaluate whether improved replicative fitness in human cells had compromised virus yield in eggs, peak TCID_50_ titres were measured for each replicate of A/SLOV15 variants during the egg time-course infections and were compared to parental V1 (Fig 2). Peak egg titres for 11 of 12 mutants were comparable to V1. Only mutant S4, carrying the single D222G mutation, showed significantly reduced egg yield, with a 10-fold reduction in titre relative to V1 (P < 0.001). Mutant S13 demonstrated a modest three-fold increase in peak egg titre relative to V1, although this was not found to be statistically significant. These data suggested that virus yield in eggs was not generally compromised by performing HA mutagenesis to optimise replicative fitness in hNEC.

### HA mutagenesis impacts immunogenicity but not antigenicity of A/SLOV15

To understand any impact of HA mutagenesis on the antigenic profile of A/SLOV15, serum immune responses and antigenic cross-reactivity of serum antibodies were assessed by two-way haemagglutination inhibition (HAI) assay. Ferret antisera was raised by vaccinating one ferret per *wt* group and two ferrets per LAIV group with 6.0–7.0 Log_10_TCID_50_ of the appropriate virus. Serum immune responses and antigenic cross-reactivity were characterised 14 days post-vaccination in three independent experiments. Test viruses included in this study were *wt* A/Michigan/45/2015 (A/MICH15), *wt* A/SLOV15, A/SLOV15 V1, A/SLOV15 S2-S5 single mutants and A/SLOV15 S13. A/MICH15 *wt* was included as the WHO-recommended A/H1N1pdm09 strain during the 2017–18 season along with the A/SLOV15 parental *wt*. Single mutants S2-S5 were included to assess individual contributions of each mutation to antigenicity and immunogenicity profiles relative to the *wt* reference strains and V1. S13 was also included in this assay due to replicating to the highest titres in hNEC and eggs. Results were expressed as geometric mean of HAI titres obtained from the three independent HAI assays.

Animals that received A/SLOV15 S2 and S4 generated comparable serum antibody titres relative to A/SLOV15 V1, showing that the N125D and D222G single mutations had minimal measurable impact on serum antibody response by HAI (Table 2). However, both animals vaccinated with A/SLOV15 S3 failed to generate any serum antibodies that were detectable by HAI, indicating that introduction of the D127E mutation in isolation negatively impacts immunogenicity in ferrets. In contrast, robust serum immune responses were observed for animals vaccinated with A/SLOV15 S5 and A/SLOV15 S13 relative to A/SLOV15 V1. This demonstrated that introduction of the single R223Q mutation, or all HA substitutions in combination, improved serum immunogenicity in ferrets, although differences in homologous titres were not found to be statistically significant.

**Table 2:** Individual point mutations are antigenically similar to *wt* reference viruses when tested by two-way HAI, but show variable immunogenicity.

	Ferret antisera													
	Anti- *wt* A/MICH15	Anti- *wt* A/SLOV15	Anti—A/SLOV15 V1		Anti- A/SLOV15 S2		Anti- A/SLOV15 S3		Anti- A/SLOV15 S4		Anti- A/SLOV15 S5		Anti- A/SLOV15 S13	
Viruses														
*wt* A/MICH15	**3413**	3413	91	91	117	59	<8	<8	309	619	2133	2133	1067	320
*wt* A/SLOV15	5120	**4267**	139	224	91	69	<8	<8	448	896	2133	1707	1067	747
A/SLOV15 V1	5120	4267	**112**	**224**	91	91	<8	<8	363	448	2133	2133	1067	427
A/SLOV15 S2	3413	2560	91	69	**69**	**91**	<8	<8	224	363	1707	2133	1707	427
A/SLOV15 S3	2560	2133	181	155	112	181	**<8**	**<8**	309	448	1707	1280	853	427
A/SLOV15 S4	2560	2560	112	91	91	91	<8	<8	**224**	**363**	2133	2133	853	267
A/SLOV15 S5	4267	4267	91	139	112	112	<8	<8	363	896	**2133**	**3413**	1280	640
A/SLOV15 S13	2560	2560	224	112	224	91	<8	<8	619	1133	1067	1280	**1280**	**640**

Summary table showing two-way HAI analyses of a panel of selected A/SLOV15 variants. A/Michigan/45/2015 (A/MICH15) *wt* reference virus was included due to being the World Health Organisation (WHO)-recommended A/H1N1pdm09 strain for the 2017–18 influenza season along with the parental *wt*, A/SLOV15. LAIV test viruses included all single mutants and A/SLOV15 S13 as the mutant with highest titre in hNEC. Ferret antisera were raised against these LAIV viruses and both *wt* reference strains. Table shows geometric mean of titres obtained from three independent two-way HAI assays and each column represents antiserum from an individual ferret, with antisera being obtained from two animals per A/SLOV15 LAIV variant, and one animal per *wt* virus. Homologous titres are bold and underlined. Homologous titres of S2, S3, S4, S5 and S13 obtained from each independent experiment were compared to V1 by one-way ANOVA with Dunn’s multiple comparison test. Differences were not found to be statistically significant and all measurable HAI titres were within 4-fold of the homologous titre.

HA mutagenesis had little impact on the antigenic profiles of the A/SLOV15 LAIV variants included in this study when assessed by two-way HAI. Despite differences in immune responses generated, antisera raised against A/SLOV15 V1, S2, S4, S5 and S13, *wt* A/SLOV15 and *wt* A/MICH15 were able to inhibit haemagglutination by all LAIV variants and both reference *wt* viruses. In all cases HAI titres were within four-fold of the relevant homologous titre and therefore considered antigenically similar. Vaccination with A/SLOV15 S3 did not produce a detectable serum antibody response in ferrets, meaning that antigenic cross-reactivity could not be determined for antisera from this variant. However, antisera raised against the other LAIV and *wt* viruses in this experiment were all able to recognise A/SLOV15 S3 LAIV virus within four-fold of homologous titre.

In addition to two-way HAI analysis of this panel of mutants, post-infection ferret antisera raised against *wt* A/MICH15 and *wt* A/SLOV15 were used to examine the antigenic profiles of all A/SLOV15 LAIV variants in a one-way HAI (Table 3). One way HAI results were expressed as the geometric mean of HAI titres obtained from three independent experiments. All A/SLOV15 LAIV variants were recognised by both reference *wt* antisera, with all HAI titres falling within four-fold of homologous titre, meaning all test viruses were considered antigenically similar. This indicated that HA mutagenesis had minimal impact on the antigenic profiles of A/SLOV15 LAIV variants when measured by HAI.

**Table 3:** Introducing N125D, D127E, D222G and R223Q in any permutation did not significantly alter antigenicity of A/SLOV15 LAIV mutants when tested by one-way HAI.

	Ferret antisera	
Viruses	Anti- *wt* A/MICH15	Anti- *wt* A/SLOV15
*wt* A/MICH15	**2133**	2133
*wt* A/SLOV15	1280	**1707**
A/SLOV15 V1	4267	1280
A/SLOV15 S2	1067	640
A/SLOV15 S3	640	640
A/SLOV15 S4	5120	2560
A/SLOV15 S5	4267	4267
A/SLOV15 S6	1280	533
A/SLOV15 S7	1067	1280
A/SLOV15 S8	2133	1280
A/SLOV15 S9	3413	1707
A/SLOV15 S10	2560	1280
A/SLOV15 S11	2560	1280
A/SLOV15 S12	1280	1280
A/SLOV15 S13	1280	1280

Summary table outlining one-way HAI analyses of all A/SLOV15 LAIV viruses. Ferret antisera were raised against *wt* A/MICH15 and *wt* A/SLOV15 reference viruses two *wt* reference strains and the full panel of A/SLOV15 mutants. Table shows the geometric mean of titres obtained from three independent one-way HAI assays. Antisera was obtained from one ferret per *wt* virus and columns represent each ferret. Homologous titres are bold and underlined. All HAI titres are within 4-fold of the homologous titre and are therefore considered to be an antigenic match as per WHO guidance.

### Serum immune responses were dependent on replicative fitness

A cross-section of A/SLOV15 variants with varying hNEC replicative fitness (V1, S3, S8, S13—Fig 1) were selected to further investigate the impact of hNEC fitness on serum immune responses in ferrets. Animals were vaccinated with a 4.0 Log_10_ FFU dose of the appropriate LAIV virus. This lower, ferret-optimised dose was previously shown to better reproduce real-world VE data [9]. Groups of 4–12 ferrets were vaccinated with A/SLOV15 V1, S3, S8 or S13 LAIV virus, or received diluent only as unvaccinated controls. Serum immune responses were then characterised 21 days post-vaccination by performing HAI assays against A/SLOV15 V1, carrying the *wt* A/SLOV15 HA sequence.

**Fig 3:**
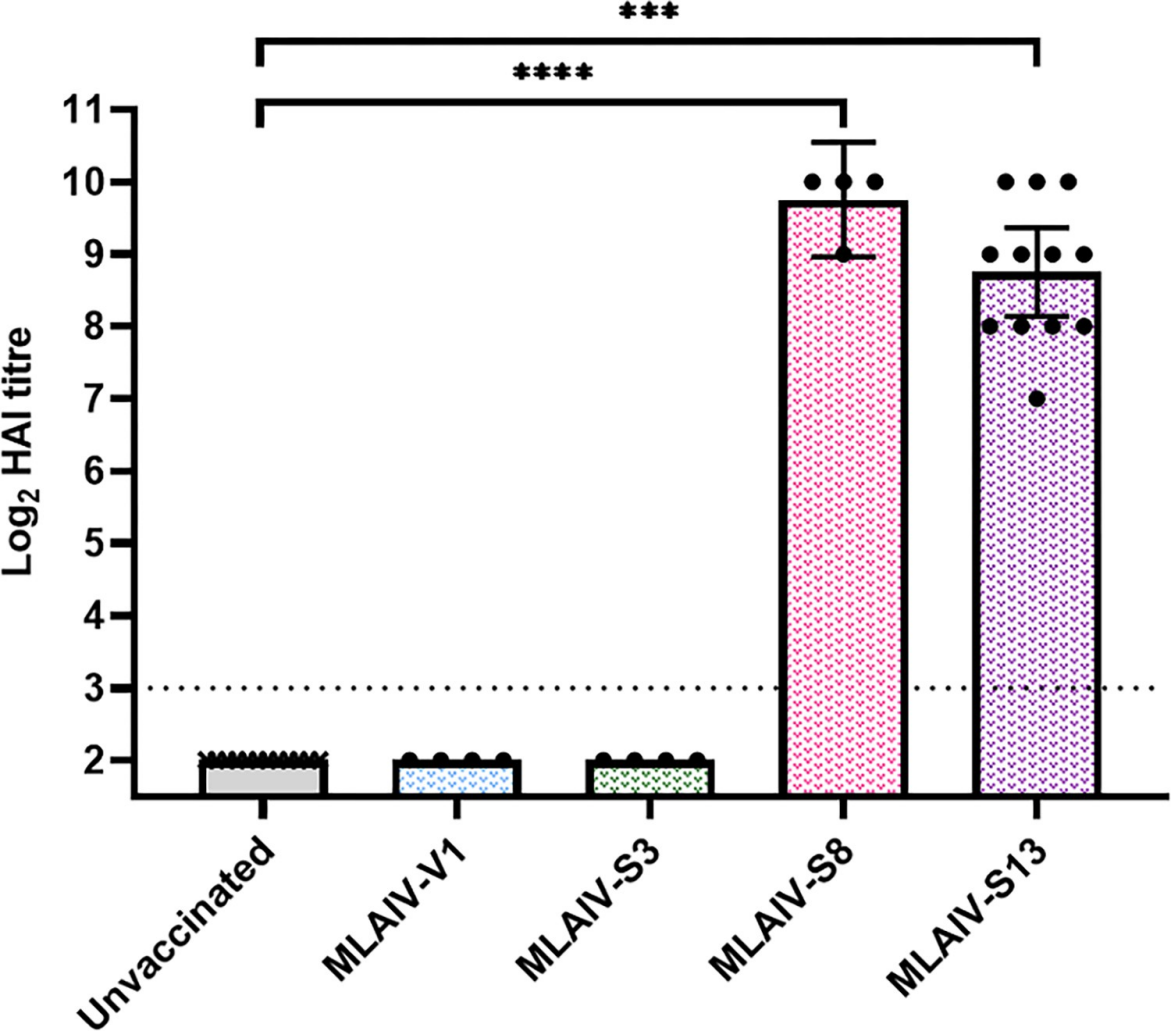
A/SLOV15 S8 and S13 elicited a serum immune response in ferrets, indicating that immunogenicity depends on replicative fitness. A cross-section of A/SLOV15 variants underwent immunogenicity testing in ferrets. Groups of 4–12 ferrets were vaccinated with 4.0 Log_10_ FFU of monovalent V1 (MLAIV-V1), monovalent S3 (MLAIV-S3), monovalent S8 (MLAIV-S8) and monovalent S13 (MLAIV-S13). Four unvaccinated ferrets were also included as controls. Serum immune responses were measured by HAI. Antisera were collected 21 days post-vaccination and titrated against parental V1 virus, containing *wt* A/SLOV15 HA sequence. MLAIV-V1, MLAIV-S3, MLAIV-S8 and MLAIV-S13 HAI titres were compared to the unvaccinated control animals by performing a Kruskal-Wallis test followed by Dunn’s multiple comparisons test. HAI titres for individual animals are depicted by the scatter plots, median values for each vaccination group are indicated by the columns. Error bars show median with 95% CI, and the dotted line represents the lower limit of detection (LLD). Values below the LLD are plotted as ½ LLD for statistical purposes. P values are indicated as follows: **** P<0.0001 and *** P<0.001.

Animals that received a low dose of A/SLOV15 V1 and S3 failed to generate a serum immune response that was detectable by HAI, and were identical to the unvaccinated group (Fig 3). In contrast, ferrets that received S8 and S13 displayed robust serum antibody titres against the *wt* A/SLOV15 HA that were significantly higher than control animals (P < 0.0001 and P < 0.001, respectively). This indicated that a sufficient level of hNEC replication was required to generate serum immune responses in ferrets in this low dose model. Given their comparable serum antibody titres, S13 was selected for further characterisation due to its superior fitness in hNEC.

### HA mutagenesis improved thermal stability of the A/SLOV15 HA protein

**Fig 4:**
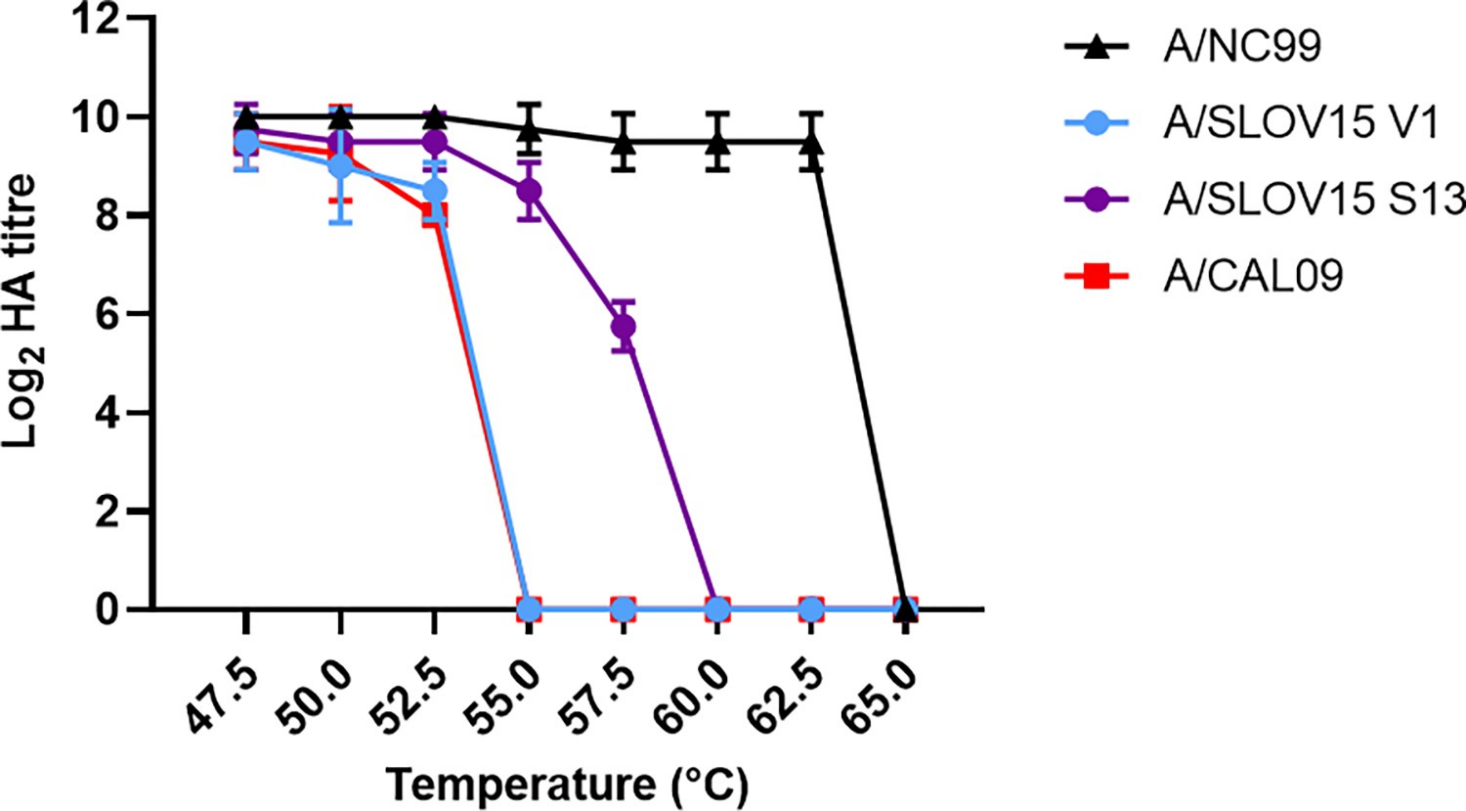
Introducing N125D, D127E, D222G and R223Q to A/SLOV15 HA protein improved thermal stability. Accelerated thermostability assay was performed with A/SLOV15 V1 (blue), A/SLOV15 S13 (purple), A/New Caledonia/20/1999 (A/NC99, black) and A/California/07/2009 (A/CA09, red) LAIV viruses. A/SLOV15 V1, A/SLOV15 S13, A/NC99 and A/CA09 viruses were held at temperatures ranging from 47.5°C—65°C for 20 minutes. After incubation, all samples underwent haemagglutination assays using 0.5% chicken red blood cells and log_2_ titres were recorded.

Reduced thermal stability of A/CA09 HA protein may have contributed to lower than expected VE estimates for this strain [22–24] during the 2013–14 influenza season. To ensure that HA mutagenesis would not impact VE for A/SLOV15, an accelerated thermostability assay was performed using V1 and S13 LAIV viruses to compare these variants with A/CA09, and a thermally stable pre-pandemic H1N1, A/New Caledonia/20/1999 (A/NC99) [22]. To be a viable LAIV CVV, S13 was required to demonstrate increased HA stability relative to A/CA09 prior to being progressed into the optimised ferret efficacy study. S13 was the only A/SLOV15 mutant included in this assay due to being the lead A/H1N1pdm09 LAIV vaccine candidate after characterisation in hNEC, eggs and HAI assays. HA thermostability of V1 was assessed alongside S13 to investigate whether mutagenesis impacted HA stability. This assay assessed the “cut-off” temperature at which the viruses were no longer able to agglutinate red blood cells. Viruses were incubated for 20 minutes at temperatures ranging between 47.5°C—65°C and HA titres were measured (Fig 4).

This accelerated thermostability assay showed that A/SLOV15 V1 and A/CA09 both lost the ability to agglutinate red blood cells at 55°C, indicating that A/SLOV15 V1 was similarly thermally unstable to A/CA09. However, HA thermostability of A/SLOV15 S13 was improved relative to A/SLOV15 V1, with this variant losing the ability to agglutinate red blood cells at 60°C. While not as stable as A/NC99, with a ‘shut-off’ temperature of 65°C, this indicated that the five amino acid changes present in S13 (Table 1), resulted in improved HA thermostability compared with V1.

### A/SLOV15 S13 provided superior protection from *wt* challenge in ferrets

**Fig 5:**
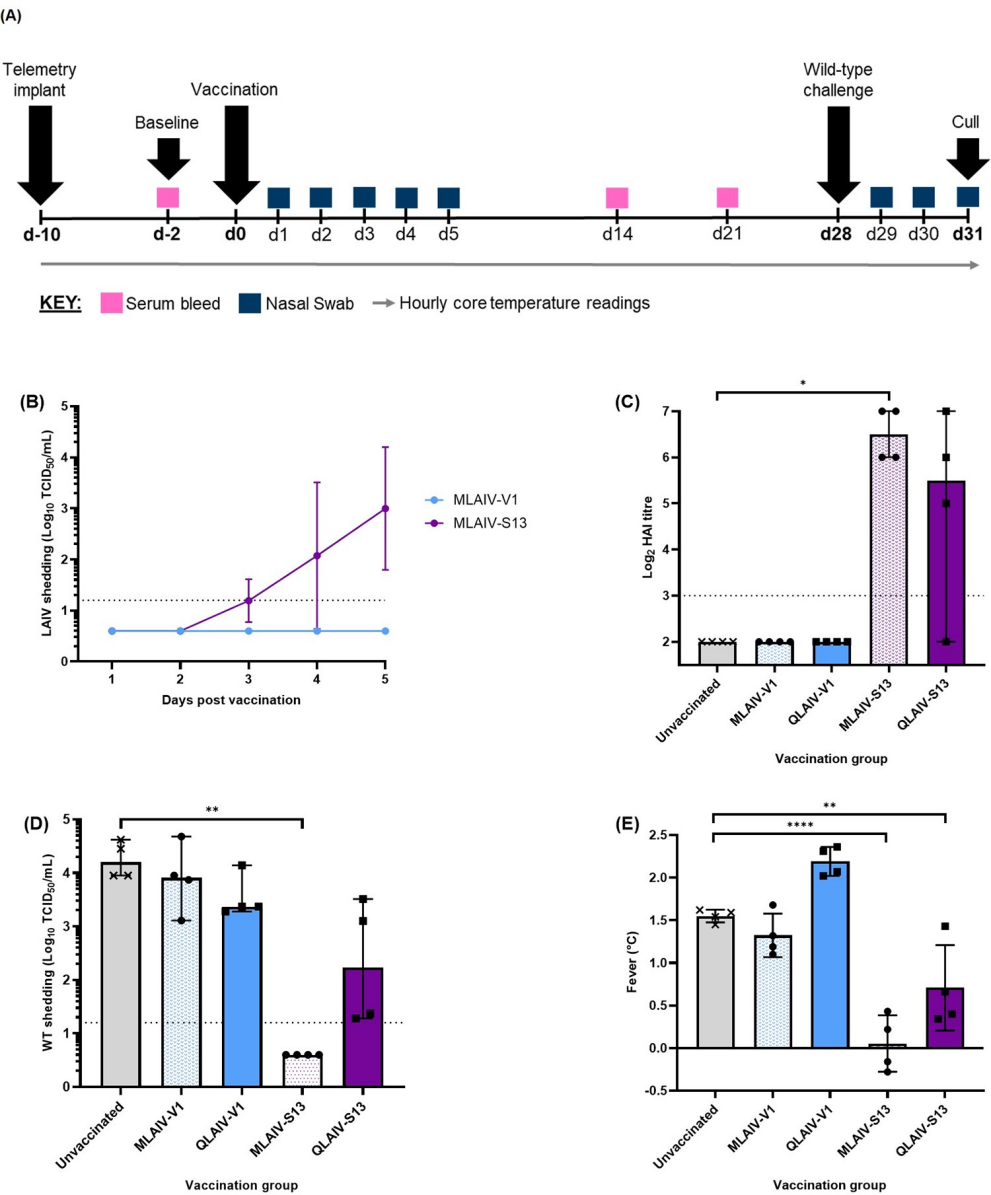
A/SLOV15 S13 gave superior LAIV shedding and serum immune responses and enhanced protection from *wt* challenge in ferrets. Groups of 4 ferrets were vaccinated with 4.0 Log_10_ FFU of monovalent V1 (MLAIV-V1), monovalent S13 (MLAIV-S13), V1 in a representative 2017–18 quadrivalent formulation (QLAIV-V1) and S13 in the same representative quadrivalent formulation (QLAIV-S13). Four unvaccinated ferrets were also included as controls. (A) Schematic of 31-day optimised ferret efficacy study plan. Days are along the horizontal axis with major interventions shown by the black arrows, coloured boxes indicating sampling points and grey arrow showing hourly core body temperature readings. (B) LAIV shedding was measured by TCID_50_ using nasal swab samples that were collected daily days 1–5 post vaccination. A/SLOV15 V1 and A/SLOV15 S13 shedding were compared by a paired t-test. (C) Serum immune responses were measured by HAI using antisera collected 21 days post-vaccination. MLAIV-V1, QLAIV-V1, MLAIV-S13 and QLAIV-S13 were compared to the unvaccinated control animals by performing a Kruskal-Wallis test followed by Dunn’s multiple comparisons test. (D) *wt* shedding was measured by TCID_50_ using nasal swab samples that were collected daily between days 1–3 post *wt* challenge and the geometric mean of *wt* shedding per day for each ferret depicted by the scatter plot. Shedding of vaccinated animals relative to unvaccinated animals was compared by a Kruskal-Wallis test followed by Dunn’s multiple comparison test. (E) Ferret core body temperature was measured hourly from pre-vaccination to study termination. Fever values as a measure of influenza-like illness for individual ferrets were obtained by subtracting temperature values recorded post-*wt* challenge from baseline temperature for each animal. Each vaccinated group was compared to the unvaccinated control group by one-way ANOVA followed by Dunnet’s multiple comparisons test. Mean values for individual animals are depicted by the scatter plots (C-E), mean values for all animals in each vaccination group are indicated by the columns (C-E), error bars show standard deviation (B,E) or median with 95% CI (C,D), and the dotted lines represent the lower limit of detection for each assay (B-D). Values below the LLD are plotted as ½ LLD for statistical purposes. P values are indicated as follows: **** P<0.0001, *** P<0.001, ** P<0.01 and * P<0.05 (B-E).

An optimised ferret efficacy study was performed to compare V1 and S13 in monovalent and quadrivalent formulations as previously described [19]. Monovalent formulations were included to compare protection provided by V1 and S13 relative to unvaccinated animals, and quadrivalent formulations indicated whether V1 and S13 were fit enough to overcome inter-strain competition in QLAIV as an indicator of VE. A minimum of ten days prior to vaccination, intraperitoneal telemetry chips were implanted to allow hourly measurements animal core body temperatures. Virus inocula included in this study were measured by fluorescent focus assay (FFA) to allow for titration of multivalent formulations, as previously described [19]. Four ferrets per study group were vaccinated with 4.0 Log_10_ FFU of the appropriate formulation. Vaccine formulations included in this study were monovalent A/SLOV15 V1 (MLAIV-V1), monovalent A/SLOV15 S13 (MLAIV-S13), A/SLOV15 V1 in a representative 2017–18 quadrivalent formulation (QLAIV-V1), A/SLOV15 S13 in a representative 2017–18 quadrivalent formulation (QLAIV-S13) and an unvaccinated group. Nasal swabs were collected daily for five days post-vaccination to measure monovalent LAIV shedding. Bleeds were collected 21 days post-vaccination to assess serum immune responses. Ferrets were then challenged with 5.0 Log_10_ FFU *wt* A/SLOV15 to assess *wt* shedding and development of fever as measures of protection from influenza-like illness (ILI). Nasal swab samples were collected for three days post-challenge to measure *wt* shedding and core body temperature data obtained from the implanted telemetry chips allowed assessment of fever (Fig 5A).

Replication and shedding of MLAIV-V1 and MLAIV-S13 *in vivo* was measured by collecting nasal swabs for five days post-vaccination and measuring infectious virus titre by TCID_50_. No A/SLOV15 V1 shedding was detectable in any animal, whereas A/SLOV15 S13 shedding was detected in all study animals: three animals 3 days post-vaccination and all four ferrets on days 4 and 5 post-vaccination (Fig 5B). This confirmed the superior replicative fitness of A/SLOV15 S13 relative to A/SLOV15 V1 *in vivo*. LAIV replication and shedding was not measured by TCID_50_ in animals that received QLAIV as TCID_50_ cannot detect individual subtypes. Measurement of shedding by qPCR was not attempted, as A/H1N1pdm09 virus shedding titres in QLAIV tend to be significantly reduced and approaching the limit of detection due to competition between subtypes in multivalent formulations. This was even seen for the highly fit and clinically effective A/NC99 strain, indicating that LAIV shedding measured by qPCR is not a useful indicator of QLAIV protection in this model [19].

Serum immune responses were assessed 21 days post-vaccination. There were no detectable antibody titres for any animals in the MLAIV-V1 and QLAIV-V1 groups when measured by HAI (Fig 5C). Conversely, MLAIV-S13 produced robust serum antibody titres of 6–7 log_2_, which was a statistically significant improvement relative to the unvaccinated group (P < 0.05). One animal in the QLAIV-S13 group failed to produce a serum antibody response, while the three remaining animals generated titres of 5–7 log_2_, comparable to MLAIV-S13. However, due to the spread of the data this improvement was not statistically significant.

Animals in each group were intranasally challenged with *wt* A/SLOV15 on day 28 post-vaccination, to assess relative levels of protection provided by V1 and S13. Shedding of *wt* virus was measured by collecting nasal swabs daily for three days post-challenge and measuring infectious virus shedding by TCID_50_. A single data point per animal was obtained for statistical comparison by calculating the geometric mean of TCID_50_ titres obtained for the three days (shown in full in S3 Fig). Again, data obtained for MLAIV-V1 and QLAIV-V1 were found to be comparable to the unvaccinated group, with no reduction in *wt* shedding observed for any animals (Fig 5D). However, vaccination with MLAIV-S13 significantly reduced *wt* shedding relative to the unvaccinated control group (P < 0.01). Two animals in the QLAIV-S13 group failed to reduce *wt* virus titre, but the two remaining animals produced *wt* shedding titres approaching the limit of detection of the assay. This indicated that two out of four ferrets vaccinated with QLAIV-S13 were protected from *wt* challenge (S3 Fig). However, overall, reduction of *wt* shedding by animals vaccinated with QLAIV-S13 was not statistically significant due to only 50% of animals being protected.

ILI was measured post-*wt* challenge by assessing development of fever in the study animals from each vaccination group, relative to unvaccinated controls (shown in full in S4 Fig). Unvaccinated animals developed significantly elevated body temperatures, with mean fever values of 1.55°C following *wt* challenge (Fig 5E). Animals in the MLAIV-V1 and QLAIV-V1 vaccination groups also developed significantly elevated body temperatures, with mean fever values of 1.32°C and 2.19°C, respectively. This corroborated data from the other endpoints in this study and suggested that all four animals in MLAIV-V1 and QLAIV-V1 groups did not respond to vaccination and were therefore not protected from ILI. In contrast, A/SLOV15 S13 provided significant reduction in fever in both the MLAIV (P < 0.0001) and QLAIV (P < 0.01) vaccination groups relative to the unvaccinated control animals, with mean fever values of 0.05°C and 0.71°C respectively. However, one animal in the QLAIV-S13 vaccination group did develop fever comparable to unvaccinated animals, reflecting what was seen for the same animal in the serum immune response and *wt* shedding endpoints of this study.

To confirm and strengthen our observations of QLAIV-S13 providing protection from *wt* challenge *in vivo*, follow up ferret studies were performed with MLAIV-S13, QLAIV-S13 and unvaccinated controls using the same study set up [19] (Fig 5A). This study demonstrated that serum immune responses were improved in animals vaccinated with QLAIV-S13 relative to unvaccinated animals (P < 0.01), with all four ferrets producing a detectable immune response when measured by HAI (S5 Fig). It was also confirmed that QLAIV-S13 provided protection from ILI as *wt* shedding was significantly reduced in three out of four animals post-challenge (P < 0.05). Development of fever was prevented in three out of four animals in the QLAIV-S13 group, which was a statistically significant improvement relative to unvaccinated ferrets (P < 0.001).

Taken together, these data suggested that A/SLOV15 V1 would not provide protection from ILI due to being unable to generate a serum immune response or prevent *wt* shedding and fever. However, data obtained from these studies indicated that S13 was of superior replicative fitness and was able to generate robust serum immune responses *in vivo* in animals vaccinated with both MLAIV and QLAIV. S13 also provided improved protection from ILI in ferrets vaccinated with S13 in monovalent and quadrivalent formulations, relative to V1 and unvaccinated animals.

### A/SLOV15 S13 shows improved binding to α-2,6 receptor analogues

**Fig 6:**
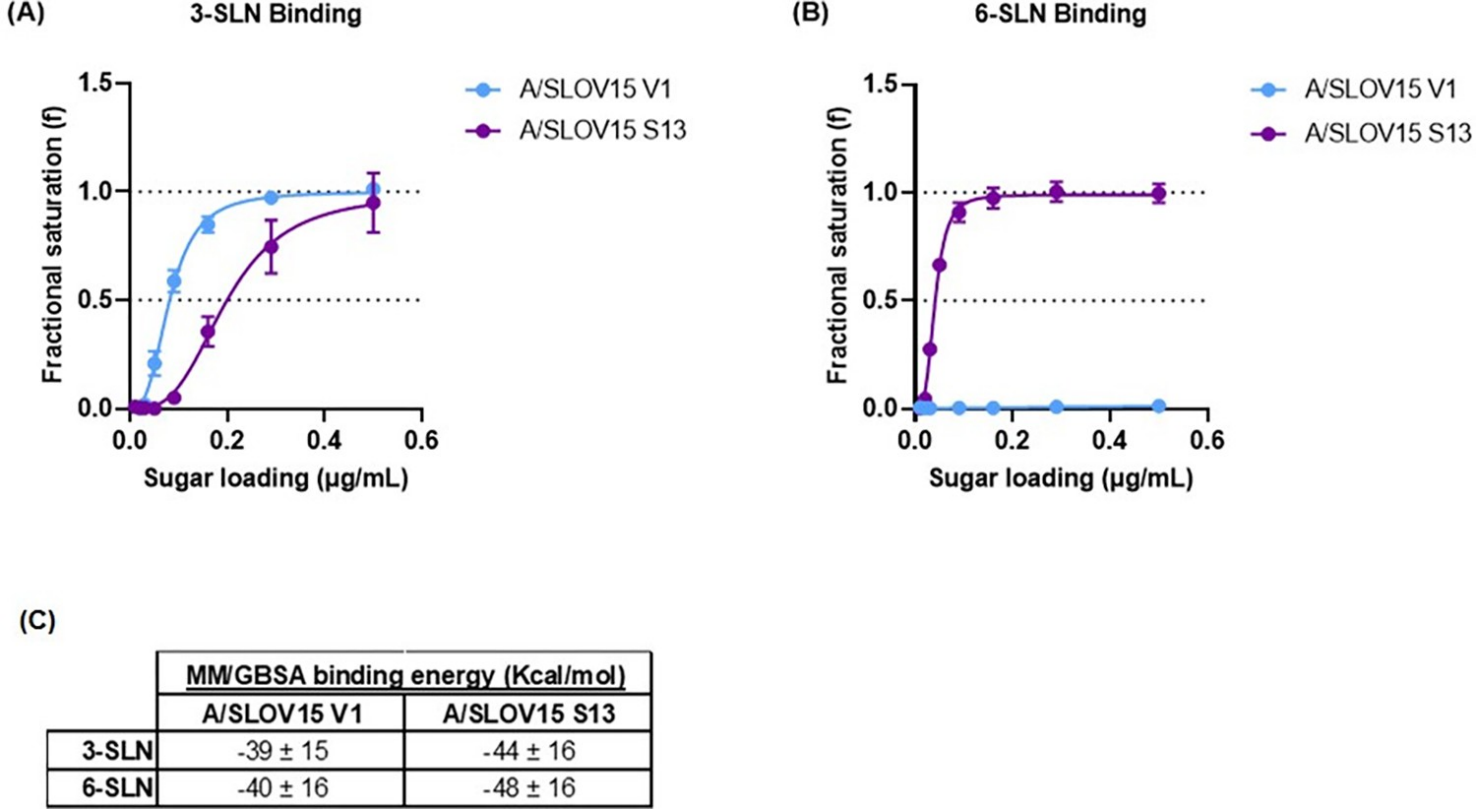
A/SLOV15 S13 displays enhanced receptor binding to α-2,6 receptor analogues relative to A/SLOV15 V1. A/SLOV15 V1 and A/SLOV15 S13 receptor analogue binding was assessed by biolayer-interferometry (BLI) using the Octet RED96 system and MM/GBSA analysis. (A,B) BLI receptor binding assays included 6.8 Log_10_ HA vRNA copies of each purified virus and binding is expressed as fractional saturation as a function of sugar loading with a best non-linear least-squares fit to the Hill equation. (A) Binding of A/SLOV15 V1 (blue) and A/SLOV15 S13 (purple) to an α-2,3 receptor analogue (3-SLN) using the Octet RED96 system. (B) Binding of A/SLOV15 V1 and A/SLOV15 S13 to an α-2,6 receptor analogue (6-SLN). (C) Binding energies of the 3-SLN and 6-SLN receptor analogues with A/SLOV15 V1 and A/SLOV15 S13 were estimated by MM/GBSA analysis (C).

It was hypothesised that the enhanced replicative fitness of S13 in hNEC and ferrets relative to V1 was due to altering the receptor binding specificity from egg-like α-2,3 receptor preference for V1, to dual α-2,3 and α-2,6 receptor specificity for S13. To investigate this, we performed biolayer interferometry (BLI) receptor binding assays on the Octet RED96 instrument. This was to determine the binding avidity of A/SLOV15 S13 relative to A/SLOV15 V1 for avian-like α-2,3 receptor analogues (3-SLN) and human-like α-2,6 receptor analogues (6-SLN). Biotinylated receptor analogues were loaded onto streptavidin biosensors at concentrations of 0.01–0.5μg/mL. Whole viruses were quantified by digital PCR (dPCR) to allow normalisation by HA vRNA content to 6.8 Log_10_ HA copies for use in these assays. Binding curves and half-saturation values were then obtained using the Hill equation to apply a non-linear least squares fit to the values obtained for fractional saturation as a function of sugar loading (Fig 6). Oseltamivir was included as a buffer component to ensure only HA protein binding was measured.

Binding data showed that A/SLOV15 S13 had slightly decreased avidity for 3-SLN relative to A/SLOV15 V1, with half saturation being reached at concentrations of 0.2 μg/mL for A/SLOV15 S13 and 0.08 μg/mL for A/SLOV15 V1 (Fig 6A). This indicated that A/SLOV15 V1 was somewhat better adapted to bind the avian receptor analogue than A/SLOV15 S13. Paired t-test of half saturation values for V1 and S13 demonstrated a statistically significant difference in binding to 3-SLN (P < 0.5). However, assessment of binding to 6-SLN revealed a dramatic difference between A/SLOV15 variants as there was no detectable binding for V1 in these assays, while S13 showed high avidity for the human receptor analogue. Paired t-test revealed S13 binding to 6-SLN was significantly increased relative to V1 (P < 0.0001), with A/SLOV15 S13 reaching half saturation at a sugar concentration of 0.04μg/mL (Fig 6B).

To expand on these *in vitro* BLI data, binding energies of the A/SLOV15 V1 and A/SLOV15 S13 HA proteins in complex with the 3-SLN and 6-SLN receptor analogues were estimated *in silico* by the molecular mechanics with generalised Born and surface area solvation (MM/GBSA) method. A/SLOV15 V1 and S13 sequences were mapped to the A/California/04/2009 crystal structure (PDB ID: 3UBE) and the 3-SLN and 6-SLN sugars were docked to measure binding energies. 3-SLN binding energies were similar for both variants (Fig 6C). However, a small increase in 6-SLN binding energy was observed for A/SLOV15 S13 HA, suggesting that it may have had increased affinity for the human receptor analogue relative to A/SLOV15 V1 HA.

Overall, these data support increased affinity and avidity of binding of S13 to the human-like 6-SLN receptor relative to V1. This confirmed that HA mutagenesis improved replicative fitness of S13 in hNEC and ferrets by altering receptor binding specificity.

### Additional interaction in A/SLOV15 S13 binding network may improve α-2,6 receptor binding

**Fig 7:**
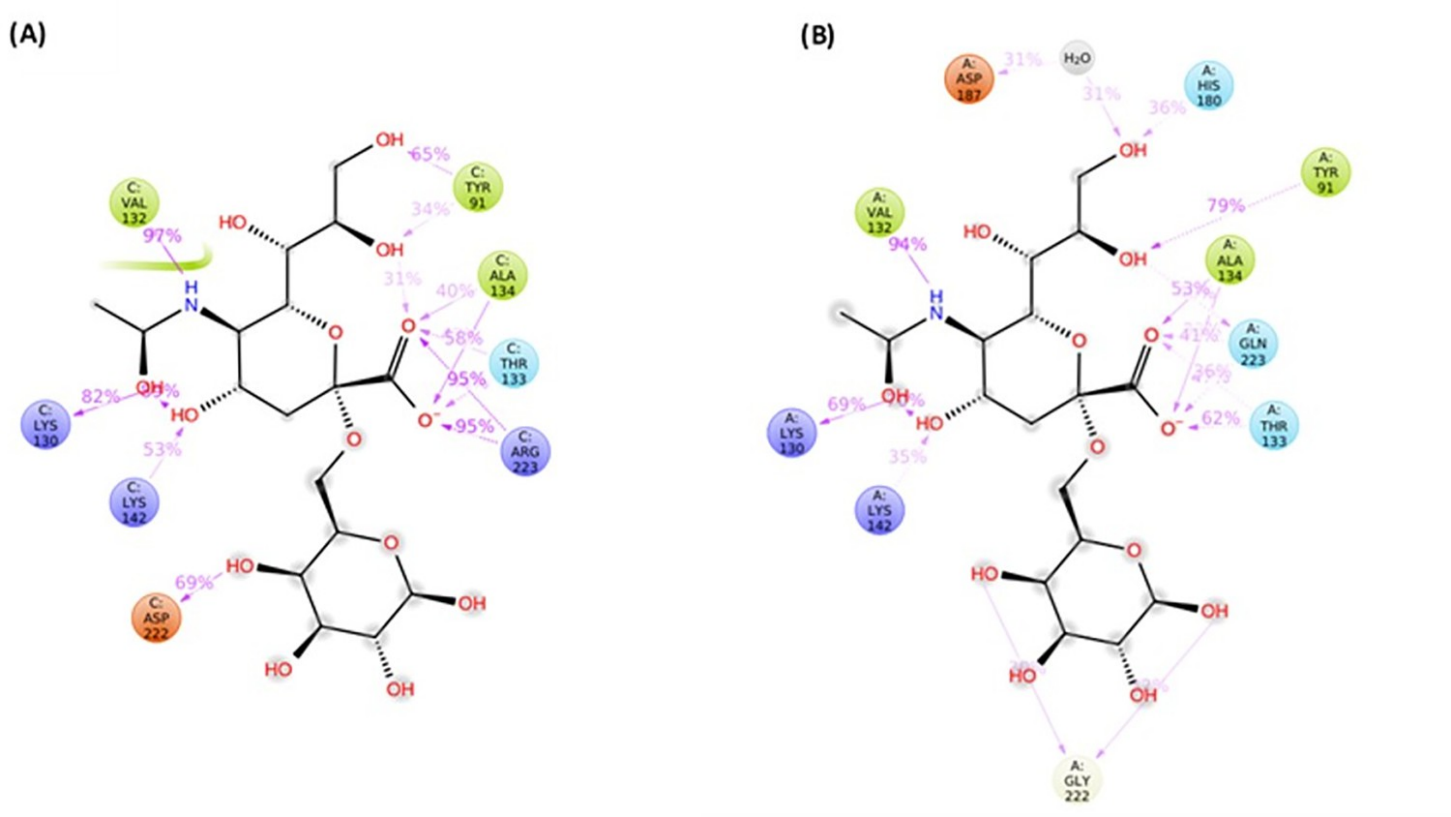
Binding network of A/SLOV15 S13 has additional interactions which may justify improved affinity to 6-SLN receptor analogue relative to A/SLOV15 V1. (A) Summary of the interactions between 6-SLN receptor analogue and A/SLOV15 V1 and (B) A/SLOV15 S13 HA proteins during molecular dynamics simulations. 6-SLN receptor analogue is shown in black and interacting receptor binding site residues are coloured by charge/polarity and mapped with percentage interaction values. Interactions that are present less than 30% of the simulation time are not shown (500ns simulation, n = 3).

Molecular dynamics simulations of A/SLOV15 V1 and A/SLOV15 S13 HA proteins binding to the 6-SLN human receptor analogue were run in Desmond via the Schrodinger suite to identify the mechanism by which A/SLOV15 S13 α-2,6 receptor binding was improved (Fig 7). The binding networks generated include interactions that were present for >30% of simulation time and show that the HA protein interactions with 6-SLN are generally well conserved between A/SLOV15 V1 (Fig 7A) and S13 (Fig 7B). However, A/SLOV15 S13 formed additional interactions, relative to V1, including D187 forming a water bridge via one of the 6-SLN hydroxyl groups, as well as an interaction with H180 via the same hydroxyl group. These additional interactions may have contributed to the improvements in 6-SLN binding affinity (Fig 6C) and avidity (Fig 6B) observed for A/SLOV15 S13 relative to A/SLOV15 V1.

## Discussion

Hawksworth *et al* previously demonstrated that A/H1N1pdm09 LAIV strains, A/CA09 and A/BOL13, replicated inefficiently in hNEC relative to the pre-pandemic H1N1 strain, A/NC99 [18]. This led to inter-strain competition in multivalent formulations and loss of efficacy *in vivo* [19], correlating with reduced VE in the clinic [17]. Here we showed that replicative fitness of an A/H1N1pdm09 LAIV CVV in hNEC could be dramatically improved by optimisation of HA protein sequence.

Combinations of N125D, D127E, D222G and R223Q HA substitutions were introduced into the egg-derived *wt* A/SLOV15 HA protein, with varying effects on replicative fitness in hNEC (Fig 1). A/SLOV15 S13, which contained N125D, D127E, D222G and R223Q alongside the naturally occurring N380D residue change, was the most improved mutant, replicating to titres 1000-fold higher than V1 during the five-day hNEC infection (Fig 1C). A/SLOV15 S12, which did not contain the naturally occurring N380D substitution, replicated to similar mean titres as S13 over 5 days in hNEC, indicating that improved replication in human cells was primarily driven by introducing N125D, D127E, D222G and R223Q to the A/SLOV15 HA protein. However, time-course data for S12 showed that virus production for this variant peaked on day 4, while S13 reached its peak titre on day 3 (S1 Fig). This suggested that N380D may also play a minor role in hNEC replication, possibly due to its position in a region of HA2 associated with fusion pH. Previously it was seen that changes in thermal stability and fusion pH tend to correlate for LAIV strains [22]. As no impact on thermal stability was seen for N380D (S7 Fig), it was considered unlikely that fusion pH would be significantly altered. However, additional investigation of pH stability could shed further light on this question.

From the perspective of selecting a CVV with improved VE, sustained virus replication in hNEC was considered beneficial. We hypothesised that the marginally more rapid replication kinetics and earlier peak of S13 (day 3) relative to S12 (day 4) could be important in overcoming inter-strain competition in QLAIV. Conversely, a mutant such as S5, which did reach a peak titre of 5.97 Log_10_ TCID_50_/mL, but replicated slowly, peaking at day 4 post-infection, could be more susceptible to inter-strain competition. This is reflected in the lower, mean five-day virus titre generated over the time-course (Fig 1). While a formal relationship between replication rate in hNEC and competition outcomes has yet to be described, this remains a subject of great interest for optimisation of LAIV development.

The D222G and R223Q residue changes, located in the RBS, likely contributed to a change in receptor binding specificity for A/SLOV15, from a preference for α-2,3 receptors to dual α-2,3 and α-2,6 specificity [20,27–29,33]. Interestingly, despite previously being described as an egg adaptation mutation [21], D127E (mutant S3) displayed the greatest increase in hNEC replication of any of the single amino acid changes tested (Fig 1A). It did not provide any significant uplift in egg yield (Fig 2), suggesting that this mutation may be more beneficial in humans than eggs. In addition, the D127E single mutation adapted A/SLOV15 for replication in hNEC to a greater extent than R223Q, which was previously shown to increase specificity to human-like α-2,6 receptors [20,27]. However, despite the D127E substitution benefitting hNEC replication, this mutation does not occur frequently in *wt* viruses [34,35]. It is possible that this is due to sequence context, with D127E enhancing replication of egg-derived LAIV reassortant viruses in human cells, but not benefiting *wt* viruses circulating in the human population. Further hNEC experiments using a panel of mutated *wt* viruses would be required to confirm this.

In contrast to the hNEC data, introduction of a D127E single mutation resulted in no detectable post-vaccination serum immune response in ferrets as measured by HAI (Table 2), while V1 and all other single mutants were immunogenic. This may have been due to subtle receptor differences between species, as the specific receptors used for replication in the upper respiratory tract have not yet been defined for either host. This indicated a possible limitation of the hNEC or ferret immunogenicity models, highlighting the importance of generating both *in vitro* and *in vivo* data during vaccine strain development.

While hNEC fitness did not correlate with immunogenicity for mutant S3, it correlated well for the remaining A/SLOV15 LAIV viruses tested. More robust serum immune responses were generated in animals vaccinated with S5 and S13, relative to V1 (Table 2). This was likely at least partly due to the R223Q substitution present in the RBS of both mutants, having previously been shown to be selected for during *wt* influenza A virus replication in the respiratory tract of ferrets [36]. Having displayed the highest hNEC replication of all mutants, S13 was also more immunogenic in ferrets. This suggested that D127E was not prohibitive to immunogenicity in combination with N125D, D222G and R223Q, which together appear to have contributed to a more robust immune response.

Measuring serum immune responses for a cross-section of A/SLOV15 mutants with varying replicative fitness in hNEC indicated that a threshold of viral fitness was required to generate serum antibodies in ferrets. Interestingly, using a more clinically translatable 4 Log_10_ FFU vaccine dose, these data suggested that immunogenicity was relatively binary. A/SLOV15 V1 and S3, with lower hNEC fitness, failed to produce a detectable response in any animals in these study groups, while fitter viruses, S8 and S13, generated comparable serum antibody titres, despite significant differences in hNEC replicative fitness. This supported a minimum level of hNEC fitness being required for immunogenicity, but also that serum antibody titres alone might not effectively differentiate between immunogenic strains.

Ferret efficacy studies using low vaccine doses were previously conducted by Dibben *et al* to investigate the low VE estimates of the A/H1N1pdm09 strain, A/BOL13 [19]. The study demonstrated that A/BOL13, which replicated relatively inefficiently in human cells, was still highly immunogenic and able to provide protection from *wt* shedding and ILI *in vivo* in monovalent formulation. However, protection was significantly reduced in trivalent and quadrivalent formulations due to competition with other sub-types [19]. This low dose efficacy animal model was applied to A/SLOV15 to determine whether V1 and S13 were able to provide protection from ILI only in their MLAIV formulations, or in both MLAIV and QLAIV.

Using a low 4.0 Log10 FFU dose, the improved fitness of S13 was clear, with no infectious virus shedding detected for MLAIV-V1, while MLAIV-S13 was increasingly detected 3–5 days post vaccination (Fig 5A). MLAIV-V1 was also unable to raise a detectable serum immune response or provide protection from *wt* virus challenge at this limited dose (Fig 5C–5E). Conversely, robust serum immune responses were observed for MLAIV-S13 and it offered significant protection against both *wt* shedding and fever. Lack of protection, even in its monovalent formulation, suggested that V1 was less effective than A/BOL13 *in vivo* [19], confirming its lack of suitability as a replacement vaccine strain. This was supported by the peak hNEC titres reached by A/BOL13 previously and V1 in this study: 6.06 and 4.09 Log_10_ TCID_50_/mL respectively [18] (S1 Fig). This reiterates the importance of using a reduced, ferret-appropriate dose to accurately identify low efficacy LAIV viruses.

In QLAIV formulation, V1 again failed to elicit detectable serum immune responses or protect ferrets from *wt* challenge (Fig 5C–5E). In contrast, ferrets vaccinated with QLAIV-S13 retained significant protection relative to unvaccinated animals, with *wt* shedding being reduced by approximately 1000-fold in 2 out of 4 animals, and fever reduced by approximately 1°C in 3 of 4 animals. QLAIV-S13 protection was confirmed in a follow-up study where it again significantly reduced *wt* shedding and ILI in both MLAIV and QLAIV groups (S5 Fig). These data indicated that optimising the hNEC replication of S13 led to superior protection *in vivo* relative to both parental V1 and the low VE A/BOL13 [19]. This builds on the work of Dibben *et al*, where differences in fitness of two antigenically distinct influenza strains, requiring different challenge viruses, were compared [19]. Here we evaluated two antigenically similar LAIV viruses, V1 and S13, with a common challenge, demonstrating that fitness alone was responsible for differences in efficacy.

It should be noted that, despite showing significant improvements, not all animals in the QLAIV-S13 vaccination group were protected, and reductions in *wt* shedding and fever were not as complete as in MLAIV-S13 animals. This indicated that even with optimised hNEC replication, a degree of competition between sub-types remained. It is also worth noting the animal-to-animal biological variations which inevitably occur during *in vivo* studies. Further studies will be required to determine the threshold of acceptable fitness for an A/H1N1pdm09 in QLAIV.

Demonstration of *in vivo* efficacy in QLAIV predicted improved clinical VE for S13, leading to its replacement of A/BOL13. Real-world VE data obtained for A/SLOV15 S13 during the 2017–18 northern hemisphere influenza season have since confirmed its effectiveness in the clinic [37], validating our approach. It should be acknowledged that VE estimates for A/SLOV15 were then reduced in the following season [38]. However, surveillance data suggested that this may have been due to antigenic drift of the A/H1N1pdm09 viruses circulating during this time [39]. The A/H3N2 and B-Victoria strain composition of QLAIV was also updated for the 2018–19 season. It is possible that A/SLOV15 S13 was differently affected by inter-strain competition in this formulation. Ferret efficacy studies using the updated 2018–19 QLAIV formulation and a representative 6B.1A challenge virus would help understand this further.

To investigate the mechanism by which fitness of S13 was improved relative to V1, receptor binding avidity was measured by BLI using whole virus. While there are multiple ways in which the HA protein can impact virus replication, here we focussed on receptor binding due to residues within and close to the RBS having undergone mutagenesis. A marginal decrease in 3-SLN binding avidity for S13 relative to V1 was shown (Fig 6A). However, this was not reflected in the egg titres obtained for these variants, with S13 replicating to slightly higher titres than V1 (Fig 2). While this increase in egg titre was not statistically significant, this highlights a key limitation of these BLI receptor binding data; a single 3-SLN receptor analogue is unlikely to be representative of the receptor diversity in an egg. Alternatively, the change in 3-SLN binding detected by BLI was too small to impact virus titre.

A vast improvement in 6-SLN binding avidity was observed for S13 relative to V1, for which there was no detectable binding to the α-2,6 receptor analogue (Fig 6B). This supported the clear improvements in hNEC replication and *in vivo* efficacy already described. This indicated that the targeted HA mutagenesis applied to S13 greatly increased its avidity to the α-2,6 receptor analogue, providing a plausible mechanism for its improved virus replication in hNEC. The maintenance of 3-SLN binding and egg yield also corroborated data published previously, indicating that 222D and 223R (V1) were associated with α-2,3 receptor specificity, while D222G and R223Q (S13) substitutions conferred dual α-2,3 and α-2,6 receptor specificity [20,27–29]. Work is ongoing to generate recombinant HA proteins and a broader range of sugars to elaborate on these data.

When binding energies between individual HA monomers and receptor analogues were predicted by MM/GBSA, a small improvement in 6-SLN affinity was observed for S13, relative to V1 (Fig 6C). This was supported by *in silico* modelling of receptor binding networks, which suggested the presence of additional interactions between 6SLN and S13: an H-bond with residue 180H and a water bridge formed by 187D, via one of the 6-SLN hydroxyl groups (Fig 7A and 7B). Due to low affinity receptor binding, multiple HA proteins must bind to gain entry into a cell during influenza virus infection [40,41]. This suggests that the small changes in 6-SLN binding affinity observed for monomeric S13 HA proteins *in silico* could account for the larger differences in avidity seen for whole virus by BLI. However, a possible limitation of this assessment is that the *in silico* modelling was performed using the A/California/04/2009 HA crystal structure (PDB ID: 3UBE); the most relevant A/H1N1pdm09 structure available when this work was conducted.

While increased α-2,6 receptor binding provides a probable mechanism for the enhanced hNEC replication of S13, the contribution of the 125/127 locus remains unclear, particularly the role of the D127E single mutation. These residues are located in close proximity to the RBS, but do not appear to be involved in 6-SLN binding (Fig 7). However, given their proximity to the RBS, it is possible that they could be playing a role in interacting with longer chain sugars not assessed here. Alternatively, the N125D and D127E substitutions may impact the pH of HA fusion due to differences in both size and side chain pKa values of these amino acids. Further work will be required to elucidate the precise contribution of N125D and D127E to improved hNEC replication.

Other relevant considerations for LAIV CVV development include egg titre, antigenicity and HA thermostability. Perhaps surprisingly, altering residues within, and in close proximity to, the RBS to improve hNEC replication did not generally compromise peak virus titres in eggs (Fig 2), contrary to existing evidence on species specificity. Peak egg titres of mutants containing 222D and 223Q residues, which have been associated with human receptor specificity [20,27], were unaffected. However, a single D222G change (mutant S4), which has previously been shown to confer dual α-2,3 and α-2,6 receptor binding specificity [20,27–29], gave a 10-fold reduction in egg yield. This may be due to the D222G substitution compromising α-2,3 receptor binding to achieve dual specificity. However, this reduction in egg yield was not observed when D222G was introduced in combination with the other residue changes investigated (S7, S9, S11-13). These data indicated that the mutations used had considerably more exaggerated effects in hNEC than eggs, suggesting a greater degree of plasticity in HA binding to egg-based receptors.

Antigenic impact of each individual point mutation was assessed by performing HAI assays. Despite residues 125 and 222 located within two of the major A/H1N1pdm09 antigenic sites, Sa and Ca respectively [26], two-way HAI data confirmed that V1, S2, S4, S5 and S13 were antigenically similar to each other and to *wt* A/SLOV15 and *wt* A/MICH15 (Table 2). This was further confirmed when all mutants were shown to be antigenically similar when tested against *wt* A/SLOV15 and *wt* A/MICH15 ferret anti-sera in a one-way HAI (Table 3). This suggested that there is sufficient flexibility in the H1 RBS to allow optimisation of receptor binding without overtly impacting antigenicity. Resolving structures of these mutant HA proteins could be valuable in understanding this further.

A/CA09 was previously shown to have an unstable HA protein which may have contributed to the reduced VE of this strain [22–24]. As a result of these findings an accelerated HA thermostability assay was introduced, which confirmed that the A/SLOV15 S13 HA protein was more thermally stable than A/CA09 (Fig 4). We hypothesised that this phenotype may have been mediated by the naturally occurring N380D residue carried by S13, which is located within a region of HA2 that has previously been associated with HA stability and fusion pH [42–44]. N380D may have improved the stability of the HA protein either due to its position at the trimer interface (S6 Fig), or by impacting the triggering of the post-fusion complex [43,45–47]. However, additional investigation demonstrated that introducing only the N380D substitution to A/SLOV15 V1 sequence did not impact thermal stability relative to V1 (S7 Fig). In addition, A/SLOV15 S12, which lacked only the N380D change relative to S13, possessed a comparable thermal stability profile to A/SLOV15 S13. Therefore, we can conclude that the N125D, D127E, D222G and R223Q mutations introduced to A/SLOV15 HA protein to enhance hNEC replication also improved its thermal stability.

This study evaluating the ability to influence VE by HA mutagenesis identified a number of key findings critical for the development of clinically effective A/H1N1pdm09 LAIV viruses. The introduction of N125D, D127E, D222G and R223Q mutations into the HA protein of A/SLOV15 greatly increased replicative fitness in hNEC via enhancement of α-2,6 sialic acid binding. Immunogenicity and HA thermostability were also improved, while not compromising antigenicity or virus yield in eggs. Crucially, enhancement of hNEC replicative fitness made A/SLOV15 S13 less susceptible to inter-strain competition, retaining efficacy in QLAIV formulation *in vivo*. This suggested that VE would be improved for S13 relative to both V1 and the previous A/H1N1pdm09 vaccine strain, A/BOL13. A/SLOV15 S13 was subsequently selected as the A/H1N1pdm09 component of the 2017–18 FluMist/Fluenz formulation and real-world VE data collected during 2017–18 influenza season confirmed clinical effectiveness [37]. This improvement in A/H1N1pdm09 VE, relative to A/CA09 and A/BOL13 during the 2013–14 and 2015–16 influenza seasons [17], validated the use of directed HA mutagenesis as a tool to rationally improve replicative fitness during strain development, allowing more clinically effective LAIV CVVs to be generated. Furthermore, this demonstrated that modifications to receptor binding had a considerably stronger effect in human cells than in eggs. This could have broader implications for studies of influenza A virus sequence evolution and species specificity.

## Materials and methods

### Ethics statement

The ferret studies described here adhere to the Home Office requirements under the Animal Scientific Procedures Act 1986 and are executed under an appropriate project license. AstraZeneca’s Council for Science & Animal Welfare (C-SAW) provided ethical approval for the ferret studies detailed below that were carried out at Charles River Laboratories (CRL) Ireland Ltd, Carrentrila, Ballina, Co. Mayo, Ireland, F26 D786. Work was conducted in compliance with the European Directive for the Protection of Animals used for Scientific Purposes, Directive 2010\63\EU, as transposed into Irish law under Statutory Instrument, S.I. No. 543 of 2012 (as amended) and under the HPRA project authorisation to carry out influenza testing in ferrets that is held by CRL (AE19108/P022). The project manager holds a valid individual authorisation from the HPRA (No. 19108\I079) and all staff were trained and hold valid HPRA individual authorisations for performing the procedures and euthanasia required by the studies.

### Cells and eggs

Madin-Darby canine kidney (MDCK) cells and HEK-293T cells were cultured and maintained in Eagle’s Minimum Essential Medium (EMEM) (BioWhittaker; Lonza; Cat. No. BE12-662F) containing non-essential amino acids, sodium pyruvate and supplemented with 10% heat-inactivated fetal bovine serum (v/v) (FBS) (Gibco; Thermo Fisher Scientific; Cat. No. 10500056), 1% penicillin-streptomycin (v/v) (Gibco; Thermo Fisher Scientific; Cat. No 15140122), and 1% 200mM L-Glutamine (v/v) (Gibco; Thermo Fisher Scientific; Cat. No. 25030018).

Fully differentiated hNEC were purchased from Epithelix (Epithelix SàRL, Switzerland) and maintained in transwells at the air-liquid interface in serum-free MucilAir culture media (Epithelix), supplemented with manufacturer recommended growth factors. Cells were incubated at 37°C and 5% CO_2_ for seven days prior to use, and basal media was replaced every 3–4 days.

Specific pathogen free embryonated hen’s eggs were obtained from Charles River Laboratories, Wilmington, USA. Embryonated hen’s eggs were incubated at 37°C with rotation and 70% humidity for 10–11 days prior to being inoculated.

### Generation of A/SLOV15 LAIV viruses

Egg-grown *wt* A/SLOV15 virus was provided by the Worldwide Influenza centre at the Francis Crick Institute, UK. The HA and NA sequences of *wt* A/SLOV15 are available on GISAID (Isolate ID: EPI_ISL_223353). A/SLOV15 HA and NA genes were amplified by RT-PCR and cloned into the pAD3000 vector as previously described [6,20]. Site directed mutagenesis of the HA gene was performed using the QuikChange lightning site-directed mutagenesis kit (Agilent technologies; Cat. No. 210518). The 6:2 reassortant A/SLOV15 viruses were generated using an 8-plasmid reverse genetics system as described previously [6,20]. Each virus carried a mutant A/SLOV15 *HA* gene segment, the *wt* A/SLOV15 *NA* gene and six internal gene segments (*PB2*, *PB1*, *PA*, *NP*, *M and NS*) from the *ca*, *ts*, *att* A/Ann Arbor/6/1960 master donor virus (MDV). A/SLOV15 LAIV mutants were propagated in the allantoic cavity of 10 to 11-day old embryonated hen’s eggs at 33°C and 70% humidity.

HA and NA sequences of A/SLOV15 viruses were verified by extracting RNA using QIAamp Viral RNA Mini Kit (Qiagen, Cat. No. 52906) as per manufacturer’s instructions. The viral RNA was amplified by RT-PCR and the resulting cDNAs underwent Sanger Sequencing. Sanger sequencing confirmed that HA sequences were as described in Table 1, NA sequences were identical to *wt* A/SLOV15 NA and there were no compensatory mutations detected in HA or NA for all mutants included in this study. A/SLOV15 stocks were also titrated by TCID_50_ assay as described below.

### Infection of primary human nasal epithelial cells

Primary hNEC were inoculated with A/SLOV15 LAIV viruses at an MOI of 0.01 TCID_50_/cell and assuming 500,000 cells per transwell. Prior to infection, the apical surface was washed with phosphate-buffered saline (PBS) pH 7.4 (Gibco; Thermo Fisher Scientific; Cat. No. 10010023) to remove accumulated mucous. 200μL of PBS was applied to the apical surface and cells were incubated at 37°C and 5% CO_2_ for 20 minutes. Apical supernatant was removed and cells were washed a further seven times with PBS. LAIV viruses were diluted to an MOI of 0.01 in PBS and 50μL/transwell was used to infect hNEC for 1 hour at 33°C and 5% CO_2_. Inocula were aspirated and cells were washed once with PBS to remove unbound virus. hNECs were incubated at 33°C and 5% CO_2_ for five days. Virus time-course samples were collected every 24 hours by adding 200μL PBS to the apical surface of each transwell and incubating for 30 minutes at 33°C and 5% CO_2_. Virus-containing apical supernatants were stored at -80°C until subsequent titration by TCID_50_ in MDCK cells, as described below.

### Infection of embryonated hen’s eggs

A/SLOV15 LAIV mutants were diluted in PBS and 10–11 day-old embryonated hen’s eggs were inoculated with 125 TCID_50_/egg. Eggs were incubated at 33°C with 70% humidity until the appropriate time-point (24h, 48h, 72h and 90h). Eggs were chilled at 4°C for a minimum of 5 hours prior to allantoic fluid being harvested and pooled within each virus group and timepoint. Time-course samples were titrated by TCID_50_, as described below, to measure replicative fitness in eggs.

### Accelerated HA thermostability

HA thermostability was determined by measuring the temperature at which test viruses were no longer able to agglutinate red blood cells (shut-off temperature). A/SLOV15 V1, A/SLOV15 S13, A/CA09 and A/NC99 were diluted to 512 HA units and incubated at 47.5°C, 50°C, 52.5°C, 55°C, 57.5°C, 60°C, 62.5°C and 65°C for 20 minutes. After incubation, HA assays were performed according to standard methods using 0.5% chicken red blood cells (Envigo, Netherlands).

### Ferret studies

Studies were conducted in outbred, mixed sex, specific pathogen free ferrets (Mustela putorius furo; Charles River Laboratories Ltd). Ferrets in the immunogenicity study were 23–30 weeks old, and ferrets in the efficacy study were 14–26 weeks old. Ferrets in both studies underwent daily health checks during the acclimatisation period, which was a minimum of seven days for healthy animals, prior to the study start dates. Once acclimatised, animals in both studies were confirmed seronegative for circulating A/H1N1pdm09 viruses by HAI assay, before being randomly assigned into groups for vaccination and housed in pairs. Once vaccinated, any adverse health observations were reported and investigated by the designated veterinarian.

### Assessment of LAIV immunogenicity in ferrets

On study day 0, two ferrets per vaccination group were lightly sedated with isoflurane (C&M Vetlink, Cat. No. OVIV030) and inoculated intranasally with 0.2mL of the appropriate A/SLOV15 LAIV virus at 6.0–7.0 Log_10_TCID_50_/dose (0.1mL per nostril).

On day 14 post-vaccination, ferrets were euthanized by intracardiac injection of 1.5 ml of sodium pentobarbitone (200 mg/ml; Chanelle Veterinary: cats.MB0022, GA0022) and blood samples were collected for analysis of serum immune responses. 45 – 90mL blood was collected per animal and allowed to coagulate before centrifuging. Serum was then decanted into 50mL falcon tubes for storage at -80°C.

### Virus inoculum titration by fluorescent focus assay (FFA)

Monovalent and quadrivalent LAIV formulations for use in ferret efficacy studies were titrated by fluorescent focus assay (FFA), to allow measurement of multiple subtypes with strain-specific antibodies, as previously described [19]. Briefly, 1:3 serial dilutions of vaccine formulations were prepared in EMEM (BioWhittaker; Lonza; Cat. No. BE12-662F) with 50 μg/ml gentamicin sulphate (Life Technologies, Cat. No. 15750–078), 2mM L-glutamine (Sigma, Cat. No. 25030081), and 0.5 μg/ml amphotericin B (Life Technologies, Cat. No. 15290018). MDCK cells were infected with 100 μL of serial dilutions and incubated at 33°C and 5% CO_2_ for 18-20h in the absence of trypsin. Plates were then fixed with 80% acetone (VWR, Cat. No.100033P) in water, and stained with subtype specific anti-HA protein primary antibodies followed by fluorescent secondary antibodies. Primary antibodies used for each subtype were as follows: A/H1N1pdm09- sheep anti-A/Michigan/45/2015 (NIBSC, Cat. No. 17/106), A/H3N2- sheep anti-A/Hong Kong/4801/2014 (NIBSC, Cat. No. 15/236), B-Vic- B/BRIS08- mouse anti-B/Victoria, C14975 (AstraZeneca), B-Yam- B/PHUK13- mouse anti-B/Yamagata, SP12-051 (AstraZeneca). Fluorescent secondary antibodies used for this assay were as follows: A strains- alexa 488 donkey anti-sheep IgG (H&L) (Life Technologies, Cat. No. A11015), B strains- alexa 488 goat anti-mouse (Life Technologies, Cat. No. A11017). Fluorescent foci were then counted using an Eclipse Ti inverted fluorescent microscope (Nikon) at x100 magnification and vaccine virus concentration was calculated and expressed as Log_10_ FFU/dose.

### Assessment of LAIV efficacy in ferrets

Ferret efficacy studies were performed as described previously [19]. Intraperitoneal telemetry chips (Data Sciences International, Cat. No. ANIPILL 0.1C) were surgically implanted a minimum of 10 days prior to vaccination to allow remote monitoring of animal body temperatures. Temperatures were read hourly from implantation to study termination.

On day 0 of the study, four ferrets per group were lightly sedated with isoflurane and intranasally vaccinated with 4.0 Log_10_FFU/strain of the appropriate A/SLOV15 LAIV formulation or a mock vaccination with the sample diluent (PBS with 1x sucrose phosphate, ThermoFisher Scientific: custom product, Cat. No. AC10210390 and 1x gelatine-arginine-glutamate, ThermoFisher Scientific: custom product, Cat. No. AC10207676). A/SLOV15 LAIV formulations were monovalent A/SLOV15 V1 (MLAIV-V1), quadrivalent A/SLOV15 V1 (QLAIV-V1), monovalent A/SLOV15 S13 (MLAIV-S13) and quadrivalent A/SLOV15 S13 (QLAIV-S13). Quadrivalent formulations consisted of the relevant A/SLOV15 LAIV variant with representative A/H3N2 (A/New Caledonia/71/2014), B-Victoria (B/Brisbane/60/2008) and B-Yamagata (B/Phuket/3073/2013) strains from the 2017–18 influenza season to simulate the FluMist/Fluenz vaccine.

Nasal swab samples were collected daily on study days 1–5 by anaesthetising the animals with an intramuscular injection of 0.1 mg/kg Medetor (Medetomidine; Chanelle Veterinary, Cat. No. PH003) and then sedating with isoflurane. A Copan FloqSwab (Copan, MINI (UTM Universal Transport Medium)) kit 1mL (medium plus pernasal flocked swab, Cat. No. 360C) was inserted and rotated in the right nostril, then eluted by light vortexing in 1mL of Copan universal transport medium before being aliquoted and stored at –80°C until subsequent measurement of virus shedding. The anaesthetic was then reversed with an intramuscular injection of 0.1 mg/kg Revertor (Chanelle Veterinary, Cat. No. PH005), a minimum of 30 minutes after the sedative.

2mL bleeds were collected from the superior vena cava on days 21 post-vaccination for the analysis of serum immune responses. On day 28 post-vaccination, ferrets were lightly sedated with isoflurane as above, before being inoculated with 5.0 Log_10_FFU/dose of A/SLOV15 cell *wt* challenge virus as a 0.2mL dose. Nasal swab samples were collected daily, as described above, for 3 days post *wt* challenge to allow subsequent measurement of *wt* shedding by TCID_50_ assay as described below. Ferrets were euthanised three days post-challenge by intracardiac injection of 1.5 ml of sodium pentobarbitone.

### Serum immune responses

HAI assays were performed by standard methods. 100μL ferret antiserum was combined with 150μL 2x receptor destroying enzyme (Deben Diagnostics, Cat. No. 370013) and incubated at 37°C for 18–20 hours. 150μL 2% (w/v) sodium citrate was then added followed by heat inactivation at 56°C for 45 minutes. Treated antiserum was diluted in PBS if necessary (required for sera raised against *wt* viruses). Two-fold dilution series of serum was then performed and 8 HAU of virus added to each well. Plates were incubated at room temperature for 30–40 minutes before addition of 0.5% chicken red blood cell suspension (Envigo, Netherlands). Plates were incubated for a further 60 minutes and HAI titres recorded as the reciprocal of the highest dilution of antiserum able to fully prevent agglutination.

### Measurement of ferret fever

The intraperitoneal telemetry chips that were implanted prior to the study monitored the core body temperatures of individual animals hourly and these data were used to identify ILI in the ferrets. Data were analysed as described previously [19], and used to predict whether each vaccine formulation was able to protect against ILI post *wt* challenge. Briefly, post *wt* challenge temperature profiles of the unvaccinated control group animals were used to define the “fever period”, which was when average body temperature was >1.5 standard deviations above the average pre-challenge baseline body temperature for each animal. Delta-temperature values were then calculated for all animals in the study by subtracting their baseline body temperature from each of their temperature values that were obtained during the fever period. The mean delta-temperature value was then calculated for each animal to show the average deviation from normal body temperature in each ferret during the “fever period”.

### Virus titration by TCID_50_ assay

Infectious virus titres were measured by TCID_50_ in MDCK cells, and expressed as Log_10_TCID_50_/mL, as previously described [48,49]. Ten-fold dilution series of virus containing samples were prepared in EMEM (BioWhittaker; Lonza; Cat. No. BE12-662F) with 1:400 10xTrypLE (Gibco; Life Technologies, Cat. No. A1217701). 96-well tissue culture plates of MDCK cells were inoculated with the dilution series and incubated at 33°C and 5% CO_2_ for 6 days. Cells were scored for cytopathic effect on an inverted light microscope and TCID_50_ titres determined by the Spearman-Karber method.

### Virus purification and HA viral RNA quantification by digital RT-PCR

LAIV viruses propagated in embryonated hen’s eggs were purified by centrifugation at 38,000 x g in a 30% sucrose cushion for 24 hours. Viruses were resuspended in PBS and stored at -80°C until use.

HA vRNA was quantified using reverse transcriptase digital PCR (RT-dPCR). Briefly, RNA was extracted from 100μL sucrose purified LAIV virus which made up to a total volume of 500μL with water before being combined with 500μL of RLT buffer and 70% ethanol. The remainder of steps were completed according to RNeasy Mini Kit (Qiagen, Cat. No. 74104) manufacturer’s instructions. Final RNA was eluted in 50μL of water.

Superscript III First Strand Synthesis System (Thermo Fisher, Cat. No. 18080051) was used to generate cDNA by following the manufacturer’s protocol. 1μL gene specific HA primer, H1_HA_742F (10μM) (GTAAAACGACGGCCAGTGAGGGGTCAAGAAGGG), which added an M13 tag to cDNA, was combined with 1μL of extracted LAIV RNA.

14.5μL dPCR reactions were then set up using 5μL cDNA at a range of 10-fold dilutions (neat, 1 x 10^−1^–1 x 10^−6^) to identify the dilution within ‘digital’ quantification range. cDNA was combined with 200nM of the M13 forward primer (GTAAAACGACGGCCAG), 800nM of the gene specific HA reverse primer (GAAATGGGAGGCTGGTG), 100nM of the gene specific hydrolysis probe (FAM/GGACACTAG/ZEN/TAGAGCCGGGA/BHQ), 7.25μL QuantStudio 3D Digital PCR Master Mix V2 (Thermo Fisher, Cat. No. A26358) and made up to the final volume of 14.5μL with water.

The dPCR reactions were loaded onto chips as described in the manufacturer’s protocol using the QuantStudio 3D Digital PCR Chip Loader with QuantStudio 3D Digital PCR 20K Chip Kit V2 (Thermo Fisher, Cat. No. A26316). Thermal cycling of chips was then performed as follows: 96°C for 10 minutes, 39 cycles of 56°C for 2 minutes, 98°C for 30 seconds and a final extension at 56°C for 2 minutes. Chips were read on the QuantStudio 3D Digital PCR instrument.

Data were analysed using the dPCR Analysis Suite Application (Thermo Fisher). Briefly, the threshold for a positive well on the FAM channel was set by calculating the upper 3^rd^ standard deviation of a no template control (NTC) chip. This threshold was applied to all the wells on each chip using the dPCR Analysis Suite Application (Thermo Fisher) and FAM positive and negative well counts were extracted. Copy numbers for HA vRNA were calculated using Poisson statistic formula described previously [50].

### BLI receptor binding assays using the Octet RED96

Polyacrylamide-linked biotinylated receptor analogues containing 20% mol sugar and 5% mol biotin, α-2,3 sialyl lactosamine (3-SLN) and α-2,6-linked sialyl lactosamine (6-SLN) were purchased from GlycoNZ (Cat. No. 0036-BP and 0997-BP, respectively). Virus binding was measured on the Octet RED96 (ForteBio) by loading 3-SLN and 6-SLN onto streptavidin coated biosensors (ForteBio, Sartorius, Cat. No. 18–5020) at 0.01–0.5μg/mL for 4 minutes in HBS-EP buffer (Cytivia, Cat. No. BR100188) with 25 μM oseltamivir acid (Insight Biotechnology, Cat. No. SC-212414) and 100μM zanamivir (Sigma-Aldrich, Cat. No. SML0492-10MG), and then virus was added at 6.8 Log_10_ HA copies/well in the same buffer. Association was measured for a duration of 30 minutes at 25°C. Blivion software (Blivion, Francis Crick Institute, London, UK) was used to plot equilibrium measurements of virus binding as a function of the concentration of sugar immobilised on the biosensor calculated from the response during the sugar loading step.

Statistical analyses were performed in GraphPad Prism 9.0 (GraphPad Software Inc, San Diego, CA, USA). Half saturation measurements were compared by paired t-test. A/SLOV15 V1–6-SLN binding was allocated a half saturation value of 0.5μg/mL, the maximum sugar loading concentration for this assay, due to no detectable binding during these experiments.

### *In silico* modelling of A/SLOV15 HA

The crystal structure of the A/H1N1pdm09 strain A/California/04/2009 HA (PDB ID: 3UBE) [33] was used as a template for A/SLOV15 HA models. The HAs were modelled using the knowledge-based method from Prime–Schroedinger [51,52]. During the modelling the ligand (a sialic acid mimetic) bound to HA was lost. The ligands were subsequently added manually, one per monomer, based on the crystal structure location.

Molecular dynamics simulations were run in Desmond, via Schroedinger suite (Schrödinger Release 2020–3: Desmond Molecular Dynamics System, D. E. Shaw Research, New York, NY, 2020. Maestro-Desmond Interoperability Tools, Schrödinger, New York, NY, 2020) [53]. The OPSL3 force field was used for the protein and ligands [54] and a SPC model for the water [55]. All systems included: 1 copy of HA (A/SLOV15 V1 or A/SLOV15 S13), 1 sialic acid ligand per monomer, ions to neutralise the system, enough water to fully solvate the simulation box. The systems were minimised and then equilibrated to a temperature of 300K and a pressure of 1 bar, following Schrodinger manual. For the production run, an MTK barostat/thermostat was used [56]. Hydrogen mass repartitioning was applied to all hydrogen atoms [57], and a timestep of 4 fs was used. The simulations were run in 3 replicates for 500ns. The total simulated time per HA monomer is 900 ns. HA is a homotrimeric copy, so each system contains three equal copies of the monomer.

Prime MM/GBSA analysis calculations were run to estimate the binding energy of the sialic acid [51,52,58], additionally the time dependent evolution of the binding site was analysed using Schroedinger suite tools.

### Statistical analysis

Data were tested for normal distribution by performing a Shapiro-Wilk test. Subsequent analyses are described in the figure legends. All graphs were generated in GraphPad Prism, version 9.0. Statistical analyses were performed in GraphPad Prism version 9.0.

## Supporting information

S1 Fig Time-course infection of A/SLOV15 mutants in primary human nasal epithelial cells. Five day time-course infections in hNEC were performed with all A/SLOV15 mutants an MOI of 0.01. Apical wash samples were collected every 24 hours and virus titre measured by TCID_50_ assay. Mean virus titre reached at each timepoint across three independent experiments (nine transwells in total) are shown for A/SLOV15 V1 and (A) A/SLOV15 single mutants; (B) A/SLOV15 double mutants and (C) A/SLOV15 mutants carrying 3–5 substitutions. Error bars indicate standard deviation.
(TIF)

S2 Fig Growth kinetics of A/SLOV15 mutants in eggs. Embryonated hens eggs were inoculated with 125 TCID_50_/egg of each A/SLOV15 mutant. Virus yield was measured by TCID_50_ assay of allantoic fluid collected 24h, 48h, 72h and 90h post-infection. Mean virus titre reached at each timepoint across three independent experiments are shown for A/SLOV15 V1 and (A) A/SLOV15 single mutants; (B) A/SLOV15 double mutants and (C) A/SLOV15 mutants carrying 3–5 substitutions. Error bars indicate standard deviation.
(TIF)

S3 Fig Shedding kinetics of *wt* A/SLOV15 post-challenge. Shedding of *wt* virus was measured on days 1–3 post challenge by TCID_50_. Graphs show *wt* virus titre measured for the four animals per study group per day, each curve represents an individual animal. Mean *wt* virus titres obtained from four unvaccinated control animals are shown with *wt* titres obtained from ferrets vaccinated with (A) MLAIV-V1; (B) QLAIV-V1; (C) MLAIV-S13 and (D) QLAIV-S13. Dotted line indicates lower limit of detection of the TCID_50_ assay. Values below the LLD are plotted as ½ LLD for statistical purposes.
(TIF)

S4 Fig Ferret core body temperature measurements post-challenge. Ferret core body temperatures were recorded hourly by intraperitoneal chips from pre-vaccination to study termination. Graphs show spline fits to the hourly temperature readings on days 1–3 post-*wt* challenge, with each temperature curve representing an individual animal. Core body temperature readings for animals vaccinated with (A) MLAIV-V1; (B) MLAIV-S13; (C) QLAIV-V1 and; (D) QLAIV-S13. (E) Core body temperatures of the unvaccinated control group during the three days post-challenge.
(TIF)

S5 Fig A/SLOV15 S13 provides protection from influenza-like illness in ferrets. Four ferrets were vaccinated with 4.0 Log_10_ FFU of either monovalent S13 (MLAIV-S13) or S13 in a representative 2017–18 quadrivalent formulation (QLAIV-S13). Eight unvaccinated ferrets were also included as controls. (A) LAIV shedding was measured by TCID_50_ using nasal swab samples that were collected daily days 1–5 post vaccination. (B) Serum immune responses were measured by HAI using antisera collected 21 days post-vaccination. MLAIV-S13 and QLAIV-S13 were compared to the unvaccinated control animals by performing a Kruskal-Wallis test followed by Dunn’s multiple comparisons test. (C) *wt* shedding was measured by TCID_50_ using nasal swab samples that were collected daily between days 1–3 post-*wt* challenge and the geometric mean of *wt* shedding per day for each ferret depicted by the scatter plot. Shedding of vaccinated animals relative to unvaccinated animals was compared by a Kruskal-Wallis test followed by Dunn’s multiple comparison test. (D) Ferret core body temperature was measured hourly from pre-vaccination to study termination. Fever values as a measure of influenza-like illness for individual ferrets were obtained by subtracting temperature values recorded post-*wt* challenge from baseline temperature for each animal. Each vaccinated group was compared to the unvaccinated control group by one-way ANOVA followed by Dunnet’s multiple comparisons test. Mean values for individual animals are depicted by the scatter plots (B-D), mean values for all animals in each vaccination group are indicated by the columns (B-D), error bars show standard deviation (A,D) or median with 95% CI (B,C), and the dotted lines represent the lower limit of detection (LLD) for each assay (B-D). Values below the LLD are plotted as ½ LLD for statistical purposes. P values are indicated as follows: **** P<0.0001, *** P<0.001, ** P<0.01 and * P<0.05 (B-E).
(TIF)

S6 Fig Positions of HA substitutions in A/SLOV15 S13. Locations of residues 125 (blue), 127 (red), 222 (yellow), 223 (green) and 380 (purple) were visualised using Pymol software (Protein Data Bank reference: 3UBE). Monomers are coloured in shades of grey and positions of HA substitutions have been highlighted in each monomer of the three-dimensional structure. Residues locations are shown from (A) a side view and (B) a top view of the HA trimer.
(TIF)

S7 Fig N380D substitution does not impact HA thermal stability. Additional accelerated thermostability assays were performed with A/SLOV15 N380D (dark blue) and A/SLOV15 S12 (light purple). A/SLOV15 V1 (light blue), A/SLOV15 S13 (dark purple), A/NC99 (black) and A/CA09 (red) LAIV viruses were included for reference. A/SLOV15 N380D, A/SLOV15 S12, A/SLOV15 V1, A/SLOV15 S13, A/NC99 and A/CA09 viruses were held at temperatures ranging from 47.5°C—65°C for 20 minutes. After incubation, all samples underwent haemagglutination assays using 0.5% chicken red blood cells and log_2_ titres were recorded.
(TIF)
