# Supplementary figures and images for: Haemagglutinin substitutions N125D, D127E, D222G and R223Q improve replicative fitness and vaccine effectiveness of an A/H1N1pdm09 live attenuated influenza vaccine virus by enhancing α-2,6 receptor binding
Source: PLoS Pathog. 2022 May 27;18(5):e1010585. doi: 10.1371/journal.ppat.1010585 (PMC9182293; doi:10.1371/journal.ppat.1010585)

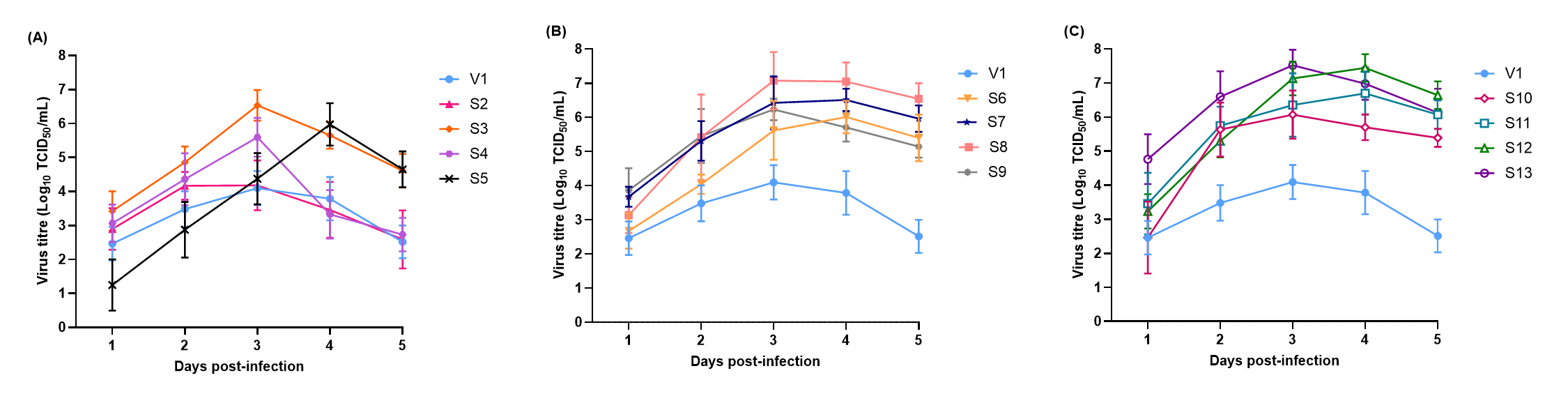

Supplement: S1 Fig — Five day time-course infections in hNEC were performed with all A/SLOV15 mutants an MOI of 0.01. Apical wash samples were collected every 24 hours and virus titre measured by TCID50 assay. Mean virus titre reached at each timepoint across three independent experiments (nine transwells in total) are shown for A/SLOV15 V1 and (A) A/SLOV15 single mutants; (B) A/SLOV15 double mutants and (C) A/SLOV15 mutants carrying 3–5 substitutions. Error bars indicate standard deviation. (TIF) [file ppat.1010585.s001.tif]

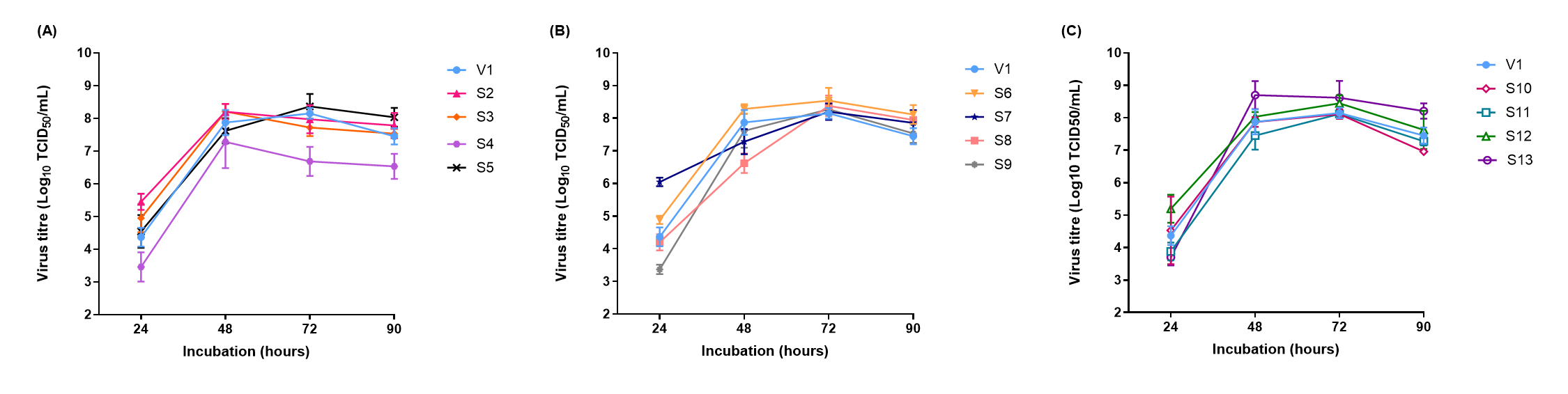

Supplement: S2 Fig — Embryonated hens eggs were inoculated with 125 TCID50/egg of each A/SLOV15 mutant. Virus yield was measured by TCID50 assay of allantoic fluid collected 24h, 48h, 72h and 90h post-infection. Mean virus titre reached at each timepoint across three independent experiments are shown for A/SLOV15 V1 and (A) A/SLOV15 single mutants; (B) A/SLOV15 double mutants and (C) A/SLOV15 mutants carrying 3–5 substitutions. Error bars indicate standard deviation. (TIF) [file ppat.1010585.s002.tif]

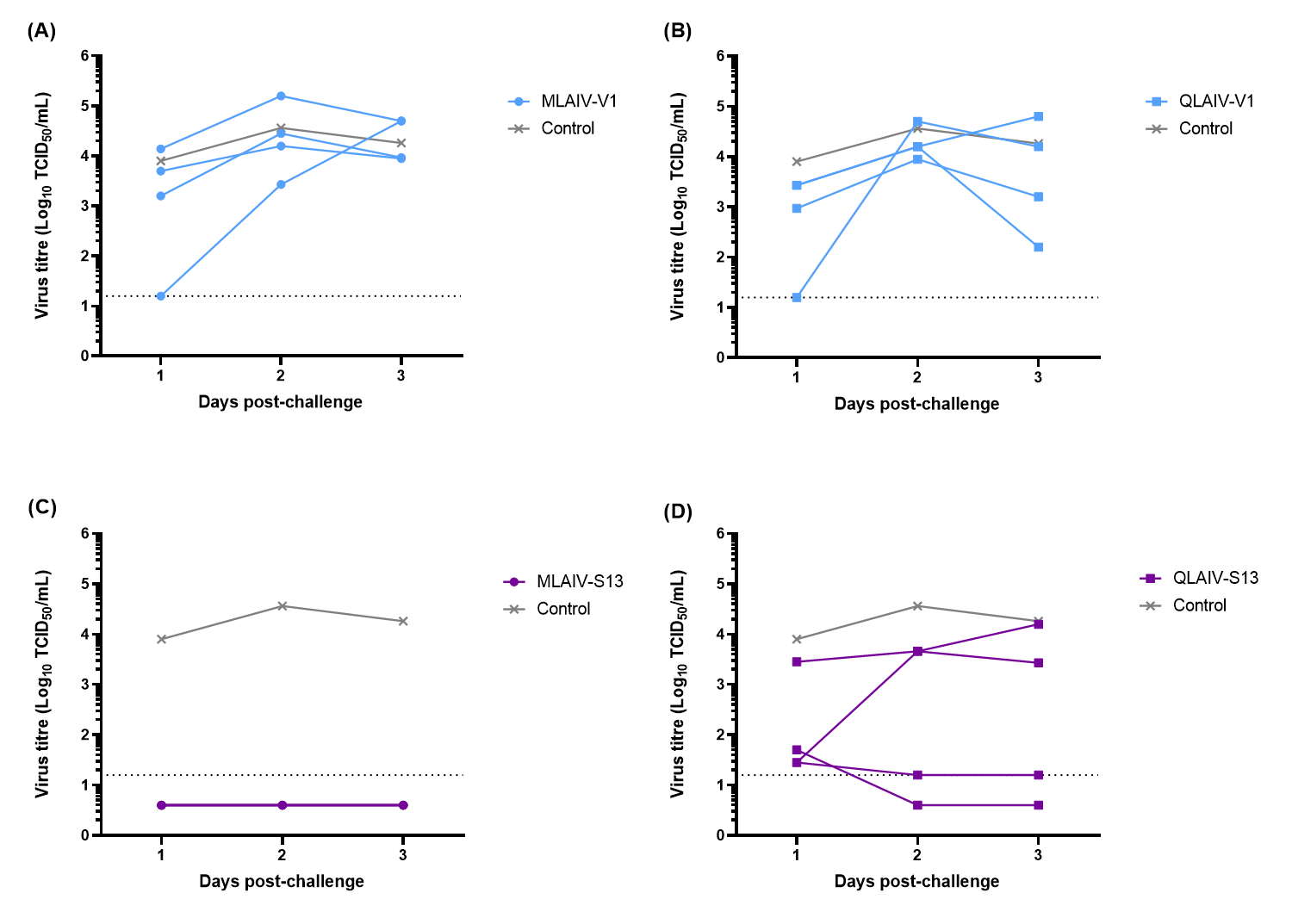

Supplement: S3 Fig — Shedding of wt virus was measured on days 1–3 post challenge by TCID50. Graphs show wt virus titre measured for the four animals per study group per day, each curve represents an individual animal. Mean wt virus titres obtained from four unvaccinated control animals are shown with wt titres obtained from ferrets vaccinated with (A) MLAIV-V1; (B) QLAIV-V1; (C) MLAIV-S13 and (D) QLAIV-S13. Dotted line indicates lower limit of detection of the TCID50 assay. Values below the LLD are plotted as ½ LLD for statistical purposes. (TIF) [file ppat.1010585.s003.tif]

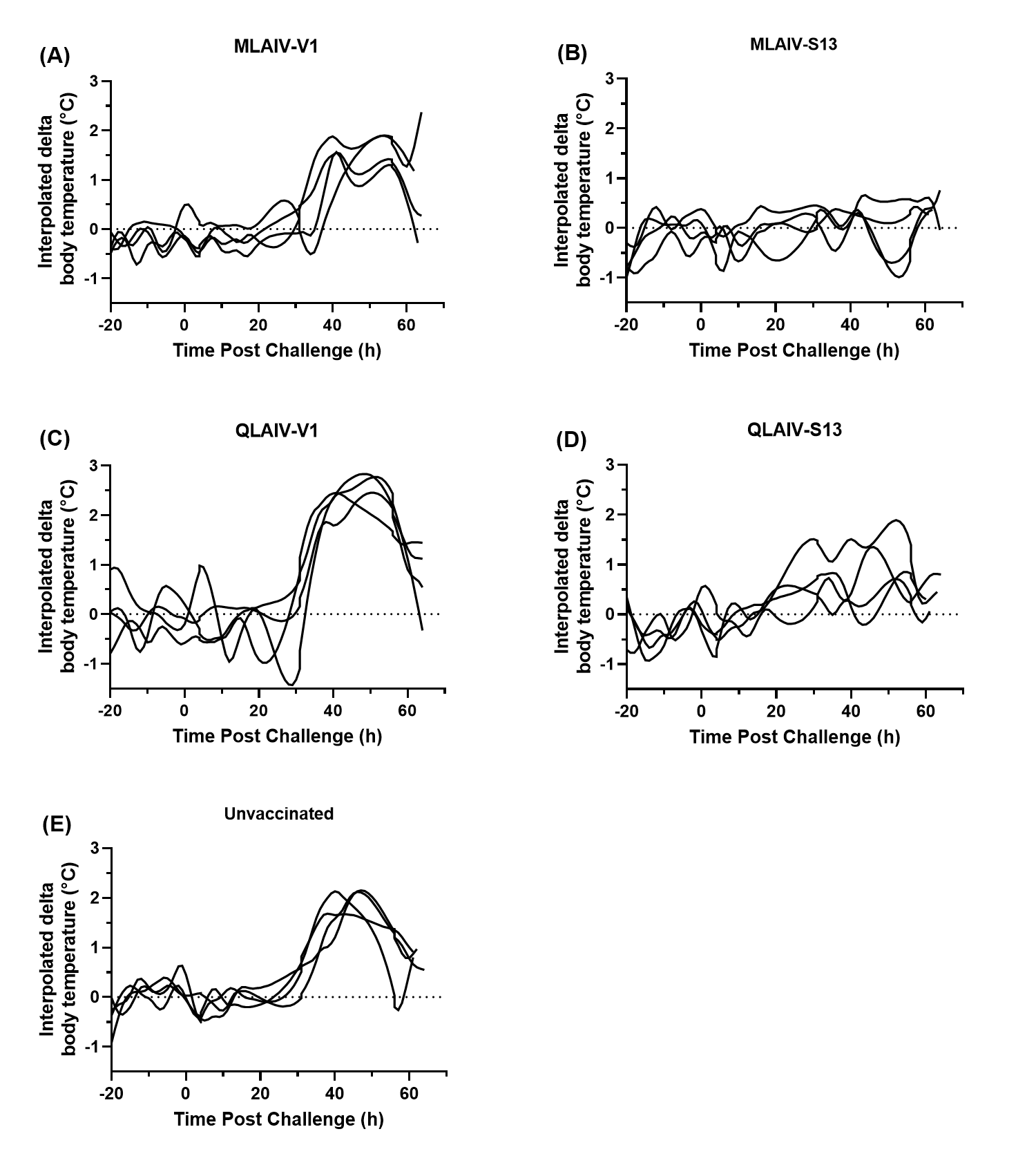

Supplement: S4 Fig — Ferret core body temperatures were recorded hourly by intraperitoneal chips from pre-vaccination to study termination. Graphs show spline fits to the hourly temperature readings on days 1–3 post-wt challenge, with each temperature curve representing an individual animal. Core body temperature readings for animals vaccinated with (A) MLAIV-V1; (B) MLAIV-S13; (C) QLAIV-V1 and; (D) QLAIV-S13. (E) Core body temperatures of the unvaccinated control group during the three days post-challenge. (TIF) [file ppat.1010585.s004.tif]

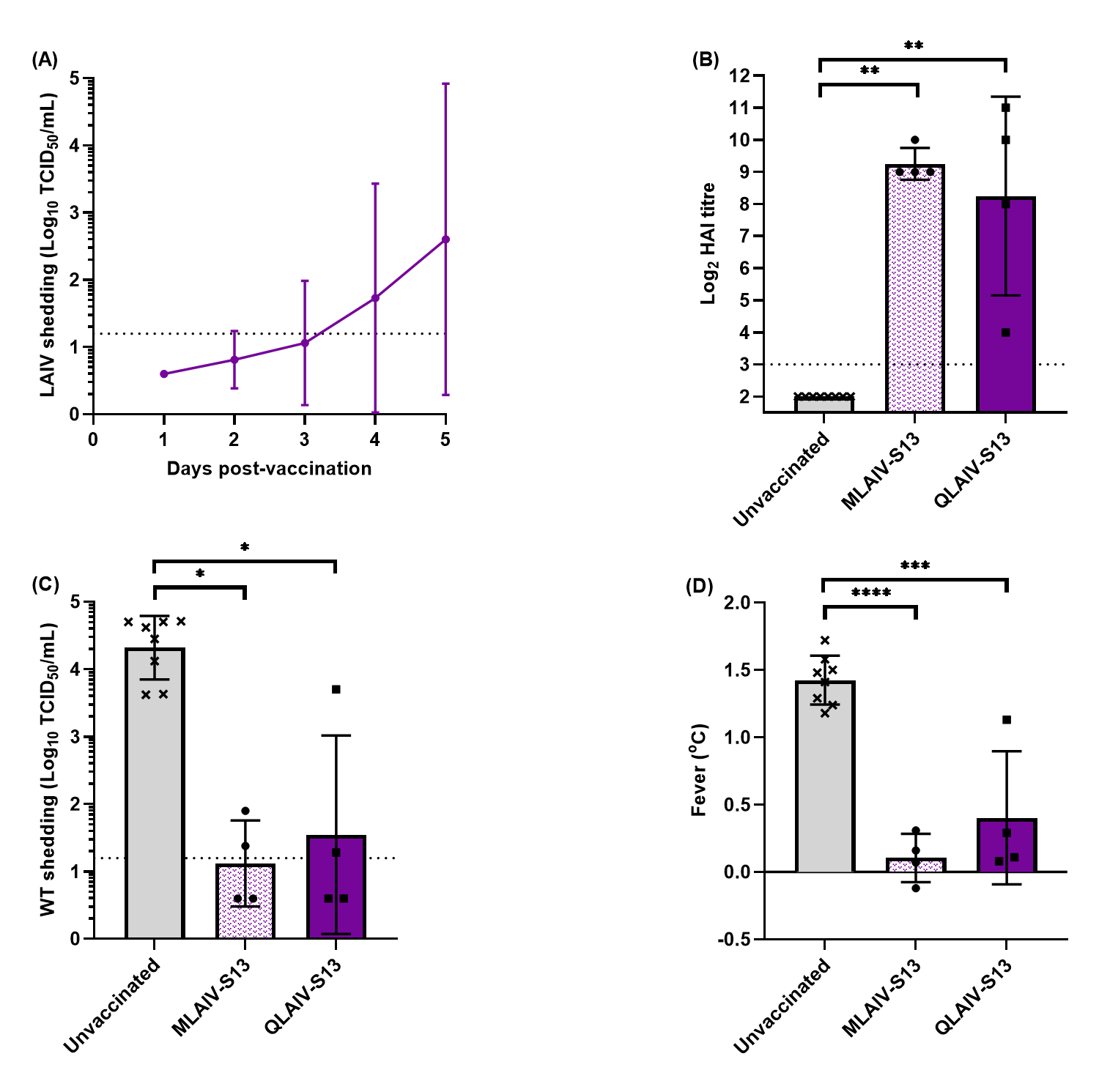

Supplement: S5 Fig — Four ferrets were vaccinated with 4.0 Log10 FFU of either monovalent S13 (MLAIV-S13) or S13 in a representative 2017–18 quadrivalent formulation (QLAIV-S13). Eight unvaccinated ferrets were also included as controls. (A) LAIV shedding was measured by TCID50 using nasal swab samples that were collected daily days 1–5 post vaccination. (B) Serum immune responses were measured by HAI using antisera collected 21 days post-vaccination. MLAIV-S13 and QLAIV-S13 were compared to the unvaccinated control animals by performing a Kruskal-Wallis test followed by Dunn’s multiple comparisons test. (C) wt shedding was measured by TCID50 using nasal swab samples that were collected daily between days 1–3 post-wt challenge and the geometric mean of wt shedding per day for each ferret depicted by the scatter plot. Shedding of vaccinated animals relative to unvaccinated animals was compared by a Kruskal-Wallis test followed by Dunn’s multiple comparison test. (D) Ferret core body temperature was measured hourly from pre-vaccination to study termination. Fever values as a measure of influenza-like illness for individual ferrets were obtained by subtracting temperature values recorded post-wt challenge from baseline temperature for each animal. Each vaccinated group was compared to the unvaccinated control group by one-way ANOVA followed by Dunnet’s multiple comparisons test. Mean values for individual animals are depicted by the scatter plots (B-D), mean values for all animals in each vaccination group are indicated by the columns (B-D), error bars show standard deviation (A,D) or median with 95% CI (B,C), and the dotted lines represent the lower limit of detection (LLD) for each assay (B-D). Values below the LLD are plotted as ½ LLD for statistical purposes. P values are indicated as follows: **** P<0.0001, *** P<0.001, ** P<0.01 and * P<0.05 (B-E). (TIF) [file ppat.1010585.s005.tif]

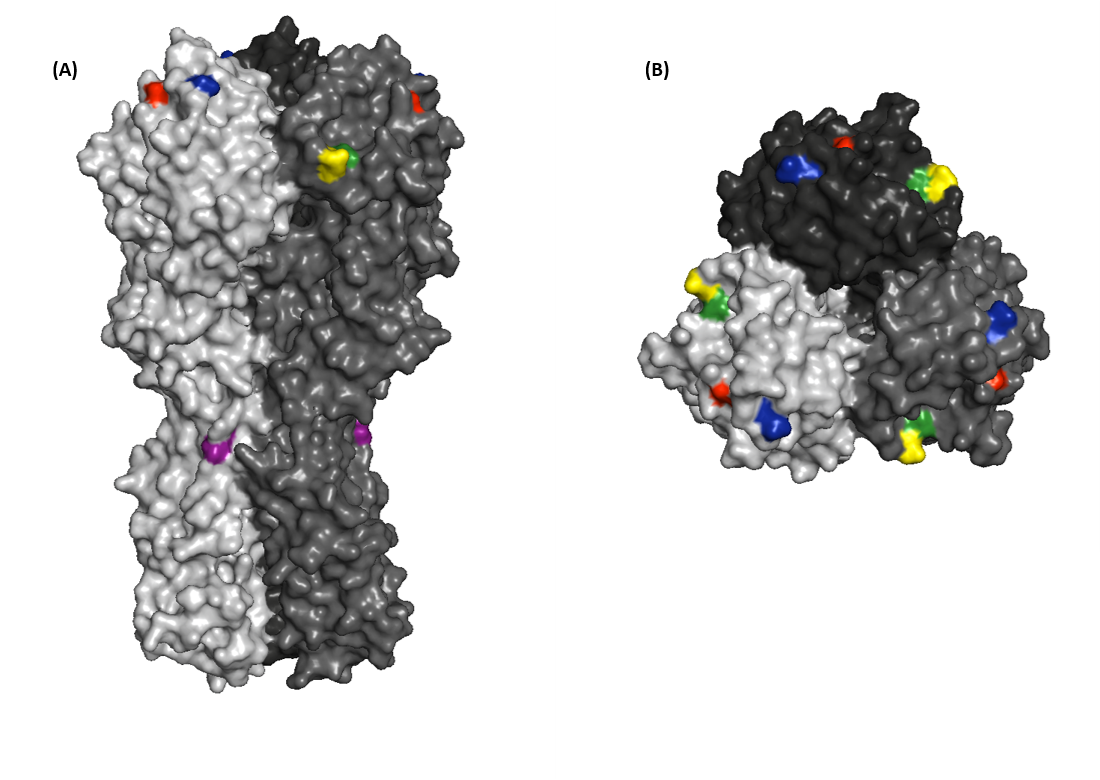

Supplement: S6 Fig — Locations of residues 125 (blue), 127 (red), 222 (yellow), 223 (green) and 380 (purple) were visualised using Pymol software (Protein Data Bank reference: 3UBE). Monomers are coloured in shades of grey and positions of HA substitutions have been highlighted in each monomer of the three-dimensional structure. Residues locations are shown from (A) a side view and (B) a top view of the HA trimer. (TIF) [file ppat.1010585.s006.tif]

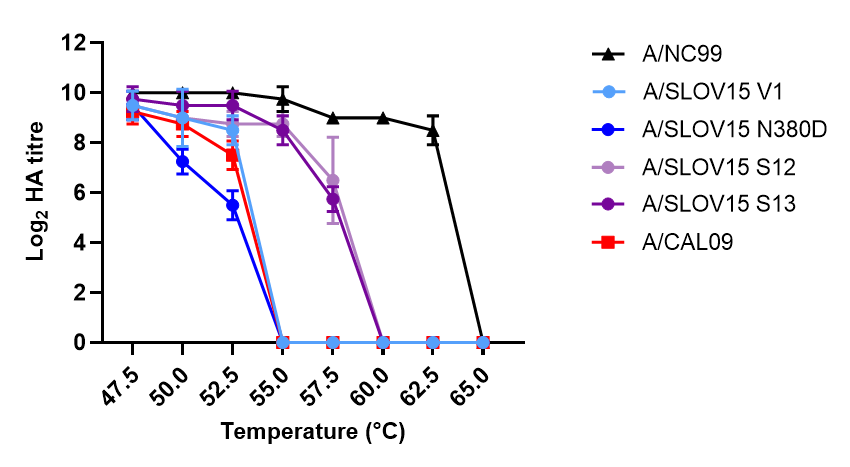

Supplement: S7 Fig — Additional accelerated thermostability assays were performed with A/SLOV15 N380D (dark blue) and A/SLOV15 S12 (light purple). A/SLOV15 V1 (light blue), A/SLOV15 S13 (dark purple), A/NC99 (black) and A/CA09 (red) LAIV viruses were included for reference. A/SLOV15 N380D, A/SLOV15 S12, A/SLOV15 V1, A/SLOV15 S13, A/NC99 and A/CA09 viruses were held at temperatures ranging from 47.5°C—65°C for 20 minutes. After incubation, all samples underwent haemagglutination assays using 0.5% chicken red blood cells and log2 titres were recorded. (TIF) [file ppat.1010585.s007.tif]
